# Application of *Pseudomonas fluorescens* to Blackberry under Field Conditions Improves Fruit Quality by Modifying Flavonoid Metabolism

**DOI:** 10.1371/journal.pone.0142639

**Published:** 2015-11-11

**Authors:** Daniel Garcia-Seco, Yang Zhang, Francisco J. Gutierrez-Mañero, Cathie Martin, Beatriz Ramos-Solano

**Affiliations:** 1 Facultad de Farmacia, Universidad CEU San Pablo, Ctra. Boadilla del Monte km 5.3, Boadilla del Monte, Madrid, Spain; 2 John Innes Centre, Norwich Research Park, Norwich, United Kingdom; South China Agricultural University, CHINA

## Abstract

Application of a plant growth promoting rhizobacterium (PGPR), *Pseudomonas fluorescens* N21.4, to roots of blackberries (*Rubus* sp.) is part of an optimised cultivation practice to improve yields and quality of fruit throughout the year in this important fruit crop. Blackberries are especially rich in flavonoids and therefore offer potential benefits for human health in prevention or amelioration of chronic diseases. However, the phenylpropanoid pathway and its regulation during ripening have not been studied in detail, in this species. PGPR may trigger flavonoid biosynthesis as part of an induced systemic response (ISR) given the important role of this pathway in plant defence, to cause increased levels of flavonoids in the fruit. We have identified structural genes encoding enzymes of the phenylpropanoid and flavonoid biosynthetic pathways catalysing the conversion of phenylalanine to the final products including flavonols, anthocyanins and catechins from blackberry, and regulatory genes likely involved in controlling the activity of pathway branches. We have also measured the major flavonols, anthocyanins and catechins at three stages during ripening. Our results demonstrate the coordinated expression of flavonoid biosynthetic genes with the accumulation of anthocyanins, catechins, and flavonols in developing fruits of blackberry. Elicitation of blackberry plants by treatment of roots with *P*.*fluorescens* N21.4, caused increased expression of some flavonoid biosynthetic genes and an accompanying increase in the concentration of selected flavonoids in fruits. Our data demonstrate the physiological mechanisms involved in the improvement of fruit quality by PGPR under field conditions, and highlight some of the genetic targets of elicitation by beneficial bacteria.

## Introduction

Flavonoids are one of the major groups of plant secondary metabolites, not only for their important functions in plants [1], but also because of their potential benefits in the diet for protection against chronic diseases [2]. Flavonoids contribute significantly to plant adaptations to changing environmental conditions, both biotic and abiotic [3,4]. Flavonoid biosynthetic pathways are strongly inducible [5] and are especially sensitive to biotic stimuli, including both fungi and bacteria [6,7].

Biotic stimuli include beneficial bacteria (plant growth promoting rhizobacteria, PGPR), some of which are able to induce plant secondary metabolism using chemical elicitors. These molecules are frequently highly conserved such that plants have been able to develop receptors for the elicitors to prevent invasion [8]; these bacterial determinants are called MAMPs (Microbe-Associated Molecular Patterns) or PAMPs (Pathogen Associated Molecular Patterns). Recognition of MAMPs, by pattern recognition receptors (PRRs), represents the first step in innate immunity, common to both plants and animals. In plant immunity, MAMPs are often general elicitors and include lipopolysaccharides (LPS), peptidoglycans, flagellin, fatty acids, sterols, proteins, which are able to elicit a defence response in hosts by binding to specific PRRs [9,10]. There are other bacterial determinants, not as conserved as MAMPs, which may overcome innate plant defence mechanisms; these are termed effectors [11].

The ability of PGPR to trigger systemic changes during plant growth has been widely demonstrated in many plant species, with the aim of increasing crop fitness/productivity [12], either by stimulating plant growth or by preventing disease [13]. Interestingly, following Induced Systemic Responses to PGPR, secondary metabolites of interest for human health may be induced. If PGPR reliably modifies secondary metabolism, then PGPR application during cultivation may provide a means of adding nutritional value to a crop and contribute to nutritional security [2]. Such treatments may therefore be of added commercial interest.

Blackberry (*Rubus spp*., *Rosaceae*) is one of the most important berries [14], with a high potential for promoting human health [15–17], and knowledge of the compounds and genes involved in the biosynthesis of phenylpropanoids in *Rubus* sp. could lead to new insights into the biological function of these compounds especially in response to elicitation by PGPR [18]. Knowledge of the activity of this pathway during ripening and in response to non-pathogenic biotic challenge should provide valuable tools for breeding and management practices to develop crops with higher quality and yield.

In pursuit of these goals, a beneficial rhizobacterium (*P*.*fluorescens*N21.4 CECT 7620) able to increase fruit yield and quality in blackberry, has been identified [19,20]. The effects of elicitation with this bacterium were studied with specific emphasis on effects on the levels of flavonoids in fruit, and changes in transcript levels of the structural genes and transcription factors of phenylpropanoid biosynthesis.

## Materials and Methods

### Plant material

*Rubus sp*. var. Loch Ness plants were grown at production fields of the company Agricola El Bosque (Lucenadel Puerto, Huelva, Spain). Plants and greenhouses were kindly provided by the company and all were handled according to regular agricultural practices [19]. Plants were grown under “winter cycle” that is, after an artificial cold period, from July to November 2012 under natural light conditions.

### Bacterial strain

The bacterial strain used was *P*. *fluorescens* N21.4 (Spanish Type Culture Collection accession number CECT 7620), a gram negative bacillus isolated from the rhizosphere of *Nicotianaglauca*. It’s able to release siderophores and chitinases *in vitro* and triggers defensive metabolism in *Solanum lycopersicum*, *Arabidopsisthaliana* [21]. It also increases isoflavone content in *Glycinemax* [22]and fruit production in *Rubus* sp.[19].

### Inoculum preparation

The bacterial strain was maintained at -80°C in nutrient broth with 20% glycerol. Inoculum was prepared by streaking strains from -80°C onto plate count agar (PCA) plates, incubating plates at 28°C for 24 h, and scraping bacterial cells off the plates into sterile 10 mM MgSO_4_ buffer. Optical density (600 nm) was adjusted to one. Inocula were delivered to plants at 10^7^c.f.u.mL^-1^.

### Experimental Design

The experimental area was defined within a blackberry production plot, arranged in three tunnel greenhouses (200 m long), each one covering two lines. Within one tunnel, one line was marked for bacterial treatments (20 plants) and another for control (20 plants). Therefore, a total of 120 plants (20 plants x 2 treatments x 3 tunnels (each tunnel was a replicate) were used in the trial.

Root inoculations were carried out by soil drench, with 500 mL of inoculum at 10^7^ c.f.u./mL in water, and watering was suspended before inoculation to prevent leakage. One week after transplantation to production greenhouses, plants were inoculated every two weeks until production finished (11 times in total). Effects on plant fitness were evaluated on growth parametres and photosynthesis. Bioactives were assessed at three ripening states (green, red and black) with colorimetric techniques, on control and bacterially-challenged plants. Specific fruit samples were taken for LC-MS-IT-ToF characterization and analysis of differential gene expression of genes in the phenylpropanoid pathways.

### Evaluation of plant fitness

#### Yield

The number of flowering stems per square meter (at the moment of maximum flowering of the plant) and number of fruits per square meter (during the production peak, 1.5 months after flowering) were recorded.

#### Chlorophyll fluorescence measurements

Chlorophyll fluorescence was measured with a pulse amplitude modulated (PAM) fluorometer (Hansatech FM2, Hansatech, Inc, UK). After dark-adaptation of leaves for 30min., the minimal fluorescence (Fo; dark adapted minimum fluorescence) was measured with weak modulated irradiation (1 μmol/m^2^ s^1^). Maximum fluorescence (Fm) was determined for the dark-adapted state by applying a 0.7 s saturating flash (10,000 μmol/m^2^ s^1^). The variable fluorescence (Fv) was calculated as the difference between the maximum fluorescence (Fm) and the minimum fluorescence (Fo). The maximum photosynthetic efficiency of photosystem II was calculated as Fv/Fm. Fluorescence emited in light conditions (Fs) and Maximum fluorescence (Fm´) were measured in a second measurement by applying a similar saturating flash after 90 s of actinic light (80 μmol/m^2^ s^1^) to produce an adaptation of leaves to light. Quantum yield PSII (ΦPSII) was calculated with the above parameters (ΦPSII = (Fm´- Fs) / Fm´) [23]. NPQ (Non-photochemical quenching) was calculated (NPQ = Fm-Fm´/Fm´) (Ögren and Baker, 1985). All measurements were carried out on10 plants for each treatment in each tunnel.

### Bioactive content characterization

For characterization of the content of bioactives, 1 g of each raw juice was extracted with 80% methanol (1/10 w/v), sonicated with an ultrasonic cleaner (Selecta 50 Hz.) for 5 min, and centrifuged at 3,500 r.p.m. for 5 min at 4°C. Colorimetric determinations were carried out on this extract as well as UPLC-IT-ToF-MS. All samples were analyzed in triplicate.

The phenolic content was determined quantitatively with Folin-Ciocalteau reagent (Sigma-Aldrich, St Louis, MO) by colorimetry [24], with modifications [20], using gallic acid as a standard (Sigma-Aldrich, St Louis, MO). Results were expressed as mg gallic acid equivalent (GAE) per 100 g of fresh weight (FW).

Total flavonol content was determined quantitatively by an aluminium chloride colorimetric assay [25] with modifications, using catechin as a standard (Sigma-Aldrich, St Louis, MO). Total flavonol content of fruit extracts was expressed as mg catechin equivalents (CE) per 100 g FW.

Total anthocyanin content was determined quantitatively by the pH differential method [26]. Results were expressed as mg of cyanidin-3-glucoside/ 100 g FW.

The radical scavenging activity of blackberry extract against the DPPH free radical (antioxidant potential) was measured using the method of Brand-Williams [27]. The antioxidant potential EC_50_ (amount of blackberry extract needed to reduce to 50% the amount of free radicals of the DPPH solution) was determined. Since EC50 is the amount of blackberry extract needed to reduce to 50% the amount of free radicals of DPPH solution, lower values indicate higher antioxidant potential.

#### UPLC-IT-ToF-MS

Samples were run on a Nexera/Prominence UPLC system attached to an Ion-Trap ToF mass spectrometer (both from Shimadzu). Separation was on a 100×2.1mm 2.7μ Kinetex XB-C18 column (Phenomenex) running the following gradient of acetonitrile (Solvent B) versus 0.1% formic acid in methanol (Solvent A) at 0.5mLmin^-1^ and 40°C: 0 min, 5% B; 12 min, 30% B; 17 min, 80% B; 17.5 min, 80% B; 17.6 min, 5% B; 21.6 min, 5% B.

Phenolics were detected by UV/visible absorbance, collecting spectra from 200-600nm, and positive electrospray MS, collecting spectra from *m/z* 220–2000. The IT-ToF also collected automatic (data-dependent) MS/MS of the most abundant precursor ions at an isolation width of *m/z* 3.0, 50% collision energy, and 50% collision gas. Precursor ions were selected not more than once per second which, combined with an overall time for carrying out all scans of 0.26 sec, means that data were collected once per second for the four most abundant ions. The instrument was calibrated according to the manufacturer’s instructions immediately before use. Spray chamber conditions were 250°C curved desorbation line, 300°C heat block, 1.5 Lmin^-1^ nebulizing gas, and drying gas “on”.

Compounds were identified and quantified by direct comparison with commercial standards (Sigma), where available, including, kaempferolrutinoside, rutin, quercetin, +(-) catechin, -(-) epicatechin and cyanidin 3-glucoside hydrate. LCMS solution version 3.0 software (Shimadzu) was used for data processing.

### RNA extraction and Quantitative Real-Time PCR

Green, red, and black fruits were sampled and frozen in liquid nitrogen. Prior to RNA extraction, samples were removed from -80°C and ground to a fine powder with liquid nitrogen using a sterilized mortar and pestle. Total RNA was isolated from green, red and black fruits with PureLink™ Micro-to-Midi Total RNA Purification System (Invitrogen^TM^). After DNase treatment and confirmation of RNA integrity using a triple check, Nanodrop™, Experion™ Automated Electrophoresis System, and electrophoresis gel, the total RNA was used for mRNA preparation, fragmentation and cDNA synthesis. Primer design was done by using online software Primer 3 Plus [28](Table 1). First strand cDNA was synthesised using SuperScript™ III (Invitrogen). One to 2 μg of total RNA (add with DEPC-treated water to 18 μL), together with 1 μL primer mix (0.25 μg/uLoligodT (Progema)) plus 0.25 μgμL^-1^ random primer (invitrogen), 1 μL 10 mM dNTP were mixed and the reaction was incubated at 65°C for 5 min and on ice for another 5 min. SIxμL 5X first-strand reaction buffer, 2 μL DTT, 1 μLRNaseOUT (invitrogen) and 1 μLSuperScript™ III were added to the mixture and incubated for 50–60 min at 50°C. The reaction was terminated at 70°C for 15 min. The cDNA product was diluted to 10 ng/μg based on the initial amount of RNA.

**Table 1:** Primers designed for RT-qPCR analysis.

Gene	Forward primer	Reverse primer
*Ru4CL*	5´CCAGAAGTTCGAGATCAACAAGT	5´GTCGGGACACTTGGTAATAGACA
*RuACT*	5´ATGTTCCCTGGTATTGCAGAC	5´CCACAACCTTGATCTTCATGC
*RuANR1*	5´TCGCAATGTACTTCCAAGAAAC	5´CTTCATCAGCTTACGGAAATCAC
*RuANR2*	5´ACTAGCTGAGAAGACAGCTTGGA	5´TGGGGATATCTGGAGTGAGACTA
*RuANS*	5´TTGGTCTGGGATTAGAAGAAAGG	5´CTGAGGGCATTTTGGGTAGTAAT
*RuC4H*	5´CATCTGTAGGGAAGTGAAGGAGA	5´ACTTCAACCCTTCGTTAGTTGTG
*RuCHI1*	5´CAAGAAGGATTCCATCATCACA	5´CTCCACTTTGATCTTTGACGACT
*RuCHI2*	5´GAGGCAGTTCTTGAGTCAATCAT	5´CACGCTATCATCACTCACTTTCA
*RuCHS*	5´ATGGTGGTTGTTGAAATTCC	5´CTGGATTGCACACCCAGGTGGCCC
*RuDFR*	5´AATCAGAAGAAGGTGAAGC	5´CATTAKSACAAGTTTGGTG
*RuF3'5'H*	5´ATGCCHCATGYYDCCTTAGCHAAAATGG	5´TGGGCAATHGGRMGAGAYCC
*RuF3H*	5´ATGGCTCCTACACCTACTAC	5´TGGATCACCGTTCAACCTGTGGAAGG
*RuF3'H*	5´CCTATCTCCAAGCTGTCATCAAG	5´GTGGTATCCGTTGATTTCACAAC
*RuFLS*	5´CCTACAGGGAAGTCAATGAGAAA	5´CACATGGGATTTCAGTACCTTCT
*RuFGT1*	5´TACTAGAATCACAAGCACCAGCA	5´ACCCAAAACTCACATAGACAACG
*RuAGT*	5´GAACTCATTGCTTGAGAGCGTAG	5´GATCTTCCACACTTCCTCTACCA
*RuLAR*	5´GTGGAGTCCCATACACGTACATT	5´CTGAAACTGATCTAACGGTGGAA
*RuPAL1*	5´GAGGAACTGGGGACTAGTTTGTT	5´AGCAGAGGATCAATCAGCTTTC
*RuPAL2*	5´GACTTGCTCTTGTTAATGGCACT	5´GAAAATCGCAGACAAGATTTCG
*RuPAT*	5´CAATGCTGCTATAAGTGTTCGAG	5´AAAAGTCCAATCAGCAGAGACTG
*RuMYB1*	5´CTCATTGACAGGAACAGGTGTC	5´CCTACAACAACACCAACGAGAAT
*RuMYB2*	5´CGATAATTGTTCGATGATGGTG	5´CTGAAATGCTGCCTCTCCTAATA
*RuMYB4*	5´ACAGCTCAGGACTCTGCTACAAC	5´GGTTTATAGACTCTTTGCCCACA
*RuMYB5*	5´ACTCAATCCAGACTCCTCATCTG	5´AGGAAGTGATTGGACTTTTAGGG
*RuMYB6*	5´TCCTATGGAGTACTTCCAAGCTC	5´TATGGCTGTTTAGTCCTCCTTGA
*RuTTG1*	5´GTTGTGATCTTGGATATCCGTTC	5´GAACAAATGTGCCTATAACTCTGC
*RuBHLH1*	5´TTTATTATACTCCGGTCGCTGGT	5´TCCTGAATCTTCTTACGGAGTTG
*RuPR1*	5´TACTACACGTACGCGACAAACAC	5´TCTCCATCATCACACACAACTCT
*RuPR2*	5´TTCGTCTCGATTATGCTCTCTTC	5´GCAGAATACACAGCATCCAAAA
*RuPR3*	5´AAATCAACCTAGCAGGCCACT	5´GAGGGAGAGGAACACCTTGACT
*RuPR4*	5´GGTGAGGATTGTTGACCAGTG	5´TCGTAGTTGACTGTAAGGTGTCC
*RuSBT1*	5´TTACAAGGTTTGCTGGAATGTG	5´GAAGACCCAATTGAGAGTGACAG

PCR reactions were undertaken by using a G-Storm Thermal Cycler (Kapa Biosystems). The reaction mixture normally consisted of 10–20 ng of DNA template, 0.1 μM each of the forward and reverse primer, 100 μM of dNTPs, 1x concentration of Taq DNA polymerase and 1x concentration Taq buffer in a total volume of 15 μL. The standard PCR protocol was as follows: initial denaturation (4 min at 94°C), followed by 25–35 cycles of denaturation (45 seconds at 94°C), annealing (30 seconds at 60°C) and extension (60 seconds at 72°C), and final extension (5 minutes at 72°C). For cloning, the PCR procedure followed was similar to that described above except for the fact that a tiny amount of bacterial colony was used to replace the template DNA. To purify DNA from PCR reactions, a QIAquick PCR Purification Kit (Qiagen) was used.

RT-qPCR analysis was performed using SYBR® Green JumpStart™ TaqReadyMix™ (Sigma). All RT-qPCRs were performed using an Opticon 2 Real Time PCR machine (MJ Research) using the following protocol: 10 min at 95°C and then 40 cycles consisting of 20 sec at 95°C, 20 sec at 60°C and 20 sec at 72°C, followed by 10 min at 72°C. Actin gene was used as reference.

To evaluate bacterial effects and their changes during ripening on all parameters measured, one-way analysis of variance was performed. When differences were significant, the least significant differences (LSD) posthoc test was also performed [29]with the software Statgraphics plus 5.1 for Windows.

## Results

### PGPR treatment increases fruit yield of blackberry

**Fig 1:**
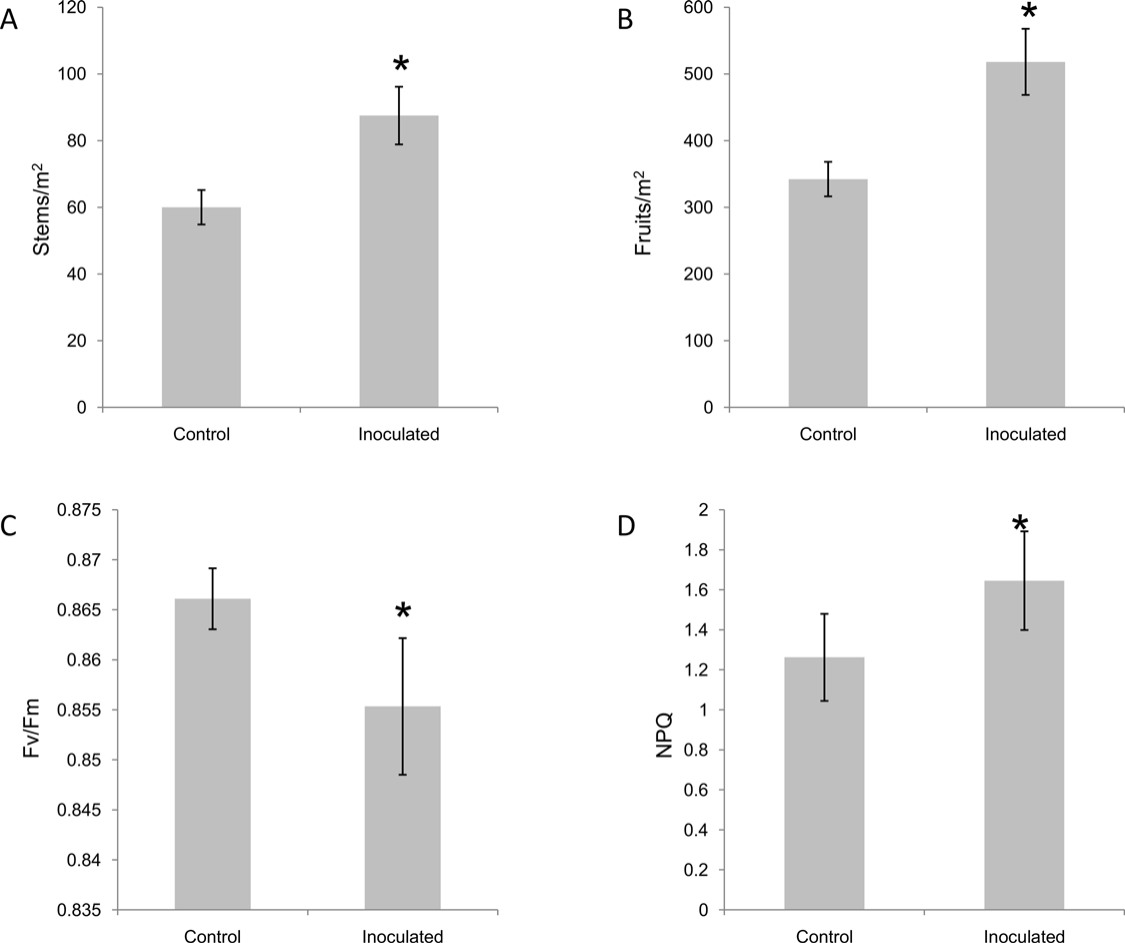
*P*.*fluorescens* increases potential fruit yield in *Rubus*sp var. Lochness. A) Fruits per m^2^ at the time of maximum flowering of the plants; B) Number of flowering stems per m^2^ during peak production, 1.5 months after flowering; C) Maximum photosynthetic efficiency of photosystem II (Fv/Fm); D) Non-photochemical quenching (NPQ)of plants inoculated with 500 mL of 10^7^c.f.u.mL^-1^ of *P*.*fluorescens* N21.4 every two weeks until production finished (11 inoculations). Data represent the mean ± SE. Lowercase letters indicate the statistical significance between different treatments according to the LSD test (*p* ≤ 0.05) (SE values are from 10 independent measurements of 60 different plants).

When *P*. *fluorescens* N21.4 was applied to *Rubus* sp. var. Loch Ness under field conditions increases in fruit yield and quality were observed [19,20]. We were interested in studying these beneficial effects in more detail. We first measured the effects of PGPR treatment on photosynthesis and yield. The number of flowering stems and fruits/m^2^ of *Rubus* sp. var. Loch Ness increased with bacterial challenge (Fig 1A and 1B) but the number of fruits per flowering stem was the same in control and treated plants. Therefore the effect of PGPR treatment was to increase the number of stems that were flowering/fruiting per unit area. Interestingly, the potential photosynthetic efficiency was significantly reduced following inoculation with *P*. *fluorescens* N21.4 (Fig 1C), and non-photochemical quenching increased under the bacterial stress (Fig 1D). Although these data were the result of single measurements of each plant significantly before harvest, they could indicate that plants treated with PGPR were stressed compared to untreated plants.

### The composition of flavonoids in blackberry fruit changes during ripening

**Fig 2:**
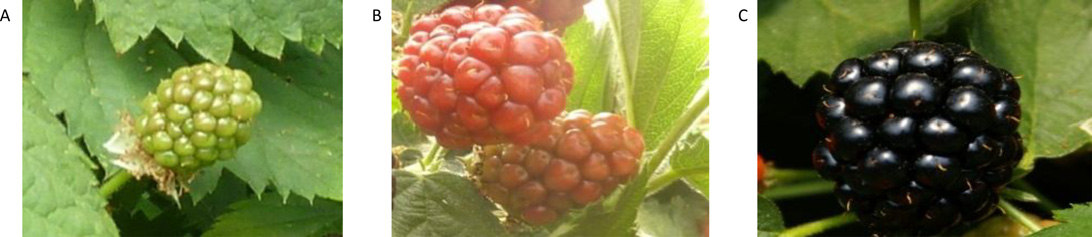
Ripening states of *Rubus* sp. var. Loch Ness. A) green, B)red, C) black.

Blackberry fruits were harvested at three main stages during fruit ripening, green fruit, red fruit and black fruit (Fig 2) from untreated plants.

**Fig 3:**
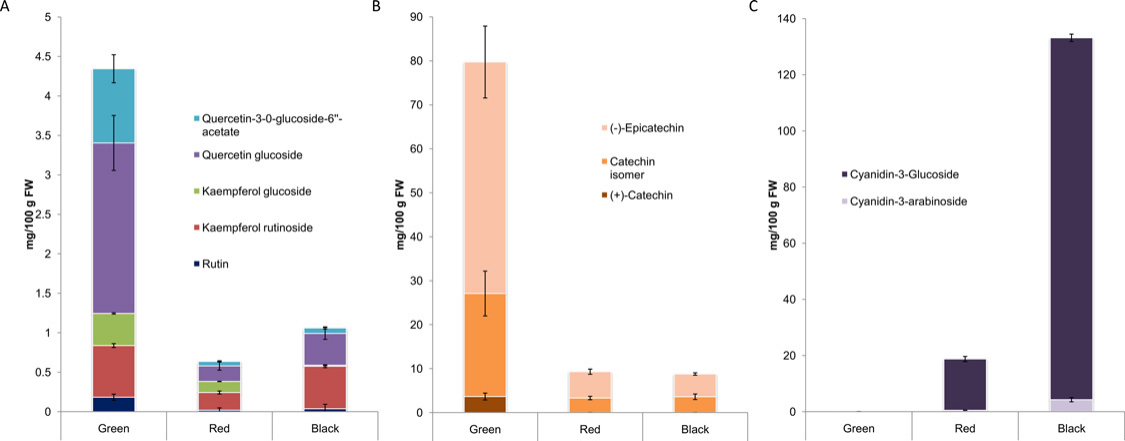
Changes in amounts of flavonols, catechins and anthocyanins in blackberry fruit during ripening. Levels of A) flavonols; B) catechins and C) anthocyanins in fruit at the three main stages of ripening; green, red and black, measured by LC-MS-IT-ToF (mg /100 gr FW). Data represent the mean ± SE. (SE values are from 3 independent measurements of 60 different plants).

LC-MS-IT was used to identify different phenylpropanoids accumulating during blackberry fruit ripening (Table 2 and Fig 3). The predominant flavonols identified and quantified in fruit were Quercetin-3-O-glucoside > Quercetin-3-O-glucoside-6''-acetate > Kaempferol-3-O-Rutinoside> Kaempferol-3-O-glucoside and Rutin (Fig 3A). Proanthocyanidins identified were catechins, (-)-epicatechin>>>, catechin isomer > (+)-catechin isomer. The two major anthocyanins, (cyanidin-3-O-glucoside > cyanidin-3-O-arabinoside) were identified only inred and black fruit (Fig 3C).

**Table 2:** Compounds identified in fruits by LC-MS-IT-ToF.

Peak	Compounds	Retention time (min)	M^+^ (m/z)
1	(+)-catechin	2.65	291.082
2	Cyainidin-3-Glucoside	2.97	449.107 (287)
3	(-)-epicatechin	3.83	291.082
4	Cyanidin-3-Arabinoside	3.82	419.096 (287)
5	Epicatechin isomer	3.64	291.082
6	Rutin	6.16	633.143 (303)
7	Kaempferol-Glucoside	6.28	449.177 (287)
8	Quercetin-glucoside	6.34	465.099(303)
9	KaempferolRutinoside	7.11	617.146 (287)
10	Quercetin-3-0-glucoside-6''-acetate	7.60	507.115(303)

The most abundant flavonols in green fruit were quercetins (quercetin-3-O-glucoside-6''-acetate and quercetin-3-O-glucoside), with 2.15 and 0.94 mg per 100g of fresh weight respectively, followed by kaempferol-3-O-rutinoside (0.65 mg per 100g FW). The highest levels of flavonols were measured in green fruit, with significant reductions in later stages of ripening (Fig 3A). For catechins, the (-)-epicatechin isomer showed the highest levels (52.6 mg per 100g FW) in green fruit, and, as with flavonols, all the catechin/epicatechin isomers showed steep declines in levels in ripening fruits (Fig 3B).Anthocyanins were absent in green fruit, but started to appear in red fruit and maximum levels were reached in mature, black fruit (Fig 3C). The main anthocyanin detected was cyanidin-3-O-glucoside (129 mg per 100g FW in ripe fruit) followed by cyanidin-3-O-arabinoside at much lower levels (4.2 mg per 100g in ripe fruit).

To investigate whether the changes in secondary metabolites during ripening were due to changes in the expression of genes encoding biosynthetic enzymes we conducted RT-qPCR to check the expression of the biosynthetic genes and regulatory proteins. To identify and quantify the expression of all genes and putative transcription factors involved in the synthesis of flavonols, anthocyanins and catechins in blackberry, sequences from the transcriptome of ripe blackberry fruit were used [30](accession number: PRJEB6680), following alignment and annotation based on previously reported genes from other Rosaceae species.

**Fig 4:**
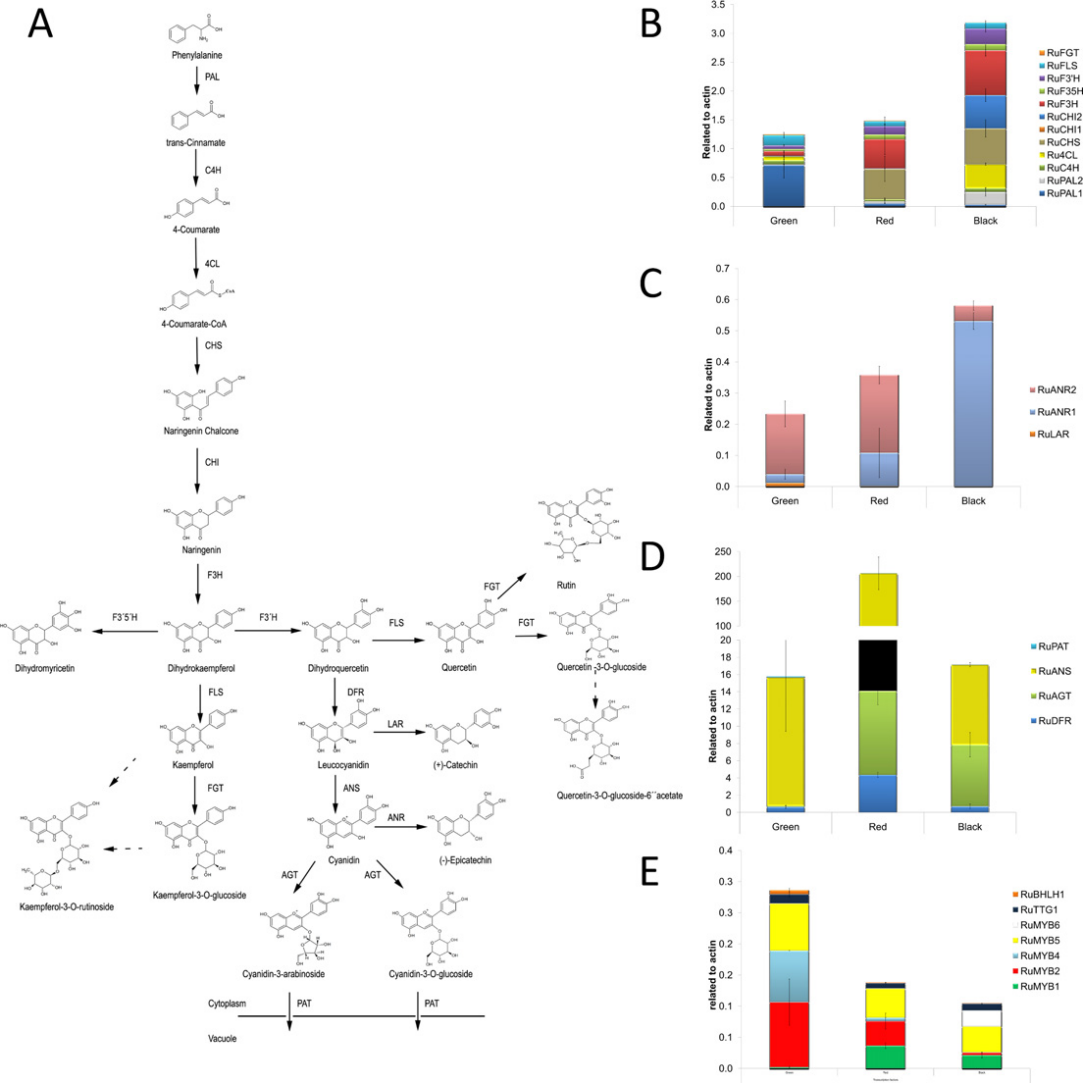
Synthesis of flavonols, and epicatechins/catechins. A)The proposed pathway of synthesis of flavonols, anthocyanins, epicatechins and catechins in *Rubus* sp. var. Lochness. Abbreviations are as follows: phenylalanine ammonia lyase (*PAL*); cinnamate-4-hydroxylase (*C4H*);4-coumaroyl-CoA-ligase (*4CL*); chalcone synthase (*CHS*); chalcone-isomerase (*CHI*); flavanone 3-hydroxylase (*F3H*);flavonoid 3´-hydroxylase (*F3*´*H*); flavonoid 3´5´-hydroxylase (*F3´5´H*); dihydroflavonol 4-reductase (*DFR*); anthocyanidin synthase (*ANS*); flavonol synthase (*FLS*); UDP-glucose: flavonoid 3-O-glucosyltransferase (*FGT*); anthocyanidin 3-O-glucosyltransferase (*AGT*); anthocyanidin reductase (*ANR*); leucoanthocyanidin reductase (*LAR*); Putative anthocyanin transporter (PAT). B; C; D; and E) Expression of genes encoding enzymes for the synthesis of flavonols (A), and epicatechins/catechins (precursors for proanthocyanidins) during ripening, and genes encoding transcriptional regulators of these pathways. Measurement of all genes was by qRT-PCR on RNA from green, red and black fruit. Data represent the mean ± SE. (SE values are from 3 independent measurements of 60 different plants).

Genes mined from the blackberry transcriptome were used as templates for primer design. All genes encoding enzymes and transcription factors involved in the phenylpropanoid pathway were searched for in the transcriptome of blackberry (S1 Table). The most highly expressed contigs encoding each candidate gene were amplified by RT-qPCR of cDNA derived from RNA extracted from fruit at each of the 3 ripening stages (Fig 4B and in more detail S1 Fig).

Since *Rubus* sp. var Lochness is a tetraploid hybrid, in the most of the cases several homologues of each gene were found (S1 Table), but there were always one or two genes with considerably higher expression than others.

Overall, the expression of genes in the general phenylpropanoid and flavonol pathways increased during ripening, although different levels of expression were measured at the three ripening stages. Expression of genes encoding enzymes of general phenylpropanoid metabolism, such as *RuPAL1*, was higher in green stage fruit, while expression of genes shared by the flavonol and anthocyanin pathways, such as *RuF3H* or chalcone isomerase (*CHI*), showed highest expression in fruit at the black stage. Interestingly, *PAL1* was highly expressed in green fruit, whereas *PAL2* was most highly expressed in black fruit. Similarly, *CHI2* showed high expression in black fruit while *CHI1* expression predominated at the earlier stages of fruit maturation. This suggested that specific isoforms of these enzymes of the general phenylpropanoid and core flavonoid pathways may be associated with specific branches (anthocyanins in black fruit, flavonols and catechins in green fruit). In the proanthocyanidin pathway, expression of *ANR2* predominated in green and red fruit, whereas *ANR1* was most highly expressed in black fruit, consistent with the changing levels of epicatechin and catechin isomers. The low expression of *LAR* in green stage fruit only was consistent with the presence of (+)-catechin and catechin isomers at this stage (Fig 4). The increase in expression of *DFR* in red fruit was consistent with the onset of anthocyanin formation at this stage, and the expression of *AGT* in red and black fruit supported a role for this protein in the transport of glycosylated anthocyanins to the vacuole at these stages.

**Fig 5:**
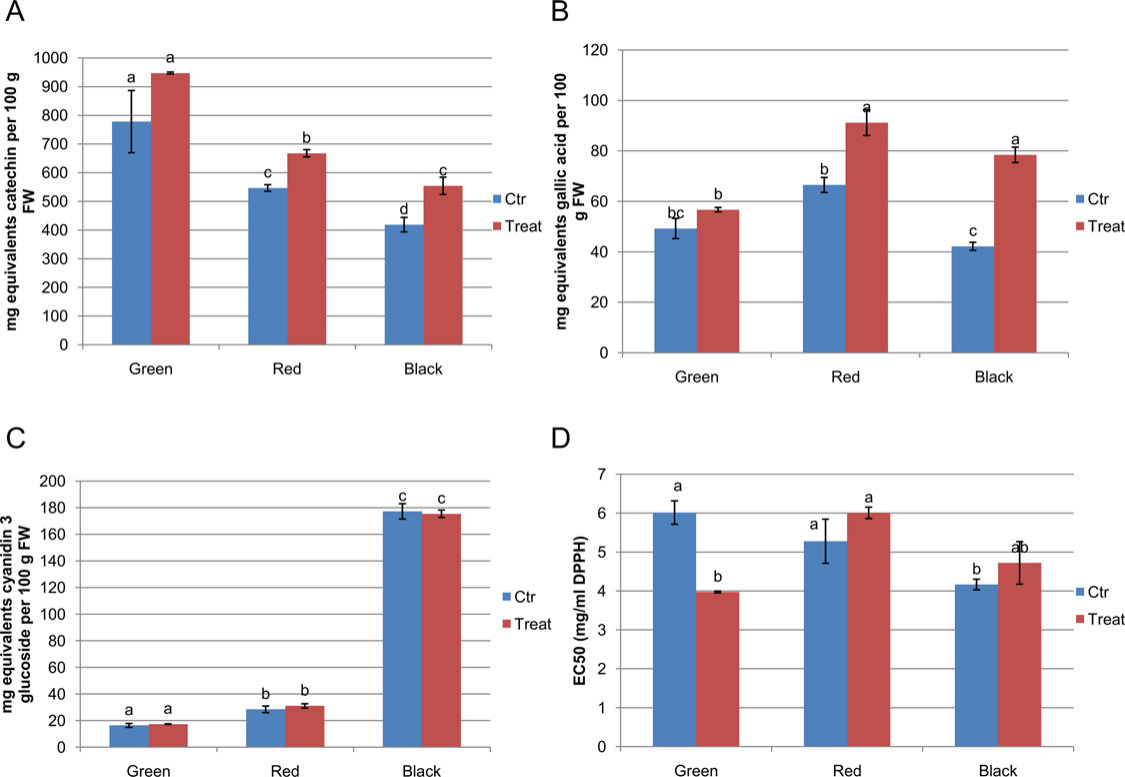
Characterisation of levels of bioactives in fruit of *Rubus* sp. var Lochness in green, red and black fruit of inoculated (500 mL of 10^7^c.f.u.mL^-1^ of *P*.*fluorescens* N21.4 every two weeks until production finished) and control plants. A) Total phenolic content expressed as mg of gallic acid equivalents (GAE) per 100 g FW; B) Total flavonoid and proanthocyanin content expressed as mg catechin equivalents (CE) per 100 g FW. C) Total anthocyanin contents expressed as mg of cyanidin-3-glucoside equivalents (CGE) per 100 g FW; D) Scavenging activities (EC50) of the DPPH radical. The EC50 values represent the volume of extract required to reduce the absorbance of the DPPH radical by half. Data represent the mean ± SE. Lowercase letters indicate the statistical significance between treatments and controls according to the LSD test (*p* ≤ 0.05) (SE values are from 3 independent measurements of fruit from 60 different plants).

Despite the high levels of expression of the genes encoding enzymes of flavonol biosynthesis (Fig 4B), flavonol concentration was lower in red or black fruits than in green fruits (Fig 5A) probably due to their immediate transformation to anthocyanins, which were accumulating at these stages (Fig 5C). The only gene specific for flavonol biosynthesis encodes flavonol synthase (FLS) and this showed lower expression in black and red fruit compared to green fruit.

Different branches of the flavonoid pathway are highly regulated at the transcriptional level, especially by MYB transcription factors [31,32]. An exhaustive search of the blackberry transcriptome for genes encoding proteins homologous to the proteins of the MYB-bHLH-WDR (MBW) complexes regulating anthocyanin and proanthocyanin biosynthesis was undertaken (S2 Fig). Expression of *RuMYB1*, encoding a homologue of *FaMYB10*, that plays a major role in the regulation of anthocyanin and phenylpropanoid metabolism during ripening of *Fragaria x ananassa* fruits [33], was higher in red and black fruit. *RuMYB2*, an homolog of *TT2*, a regulator of proanthocyanin biosynthesis [34], was highly expressed in green fruit, consistent with the higher epicatechin/catechin contents at this stage. *RuMYB4* encodes a homologue of *AtMYB4*, an inhibitor of *C4H* expression in *Arabidopsis* [35,36], and showed high expression in green fruit but disappeared in fruit at red and black stages. *RuMYB5* is homologous to *AtMYB5* in *Arabidopsis*, and the *AtMYB5* protein interacts with *TTG1* and *TT2*, two important transcription factors regulating the proanthocyanin biosynthetic pathway [37]. Expression of the *RuMYB5* gene decreased during ripening. *RuMYB6* encodes a homologue of *GmMYB12B2* from *G*. *max*, a transcription factor that activates *CHS* in soybean and *Arabidopsis*[38]; In fact, *GmMYB12B2*, induces expression of *PAL*, *CHS* and *FLS* in *Arabidopsis*[38], suggesting that it might be another regulator of flavonol biosynthesis. Expression of *RuMYB6* increased in black fruit.

*RuTTG1* encodes a homologue of *TTG1*in *Arabidopsis*, and *RuBHLH1* encodes a homologue of *TT8*, that synergistically and specifically trigger the synthesis of proanthocyanins and anthocyanins as components of the MBW complexes in *A*. *thaliana* [39]; the expression of *RuTTG1* was slightly higher in green fruit of blackberry than in later ripening stages, although the expression of *RuBHLH1* was consistently low in fruit at all stages.

### PGPR treatment results in higher levels of total phenylpropanoids, but not in total anthocyanins, in mature fruit

Total phenolics, total flavonols, total anthocyanins and antioxidant potential were measured in green, red and black fruit from plants treated with *P*.*fluorescens*N21.4 and untreated controls. Levels of total phenolics decreased during ripening, but they declined less strongly in PGPR-treated plants so that total phenolics were significantly higher in ripe black fruit of treated plants than in those of untreated controls (Fig 5A). While the levels of anthocyanins increased dramatically in red and black fruit compared to green fruit, PGPR treatment had no effect on the final levels of these compounds (Fig 5C). Levels of flavonols and epicatechins/catechins peaked in red fruit, and PGPR treatment elevated levels of flavonols and epicatechins/catechins in both green and red fruit, meaning that levels were significantly higher in treated ripe fruit than in ripe fruit of control plants (Fig 5B). EC50 values decreased from red to black stages, revealing a higher antioxidant potential in mature fruits. This was similar in control and bacterial challenged plants; however, antioxidant potential of challenged green fruits was as high as black fruits.

### *P*. *fluorescens* treatment increased expression of phenylpropanoid biosynthetic genes in the early stages of ripening

**Fig 6:**
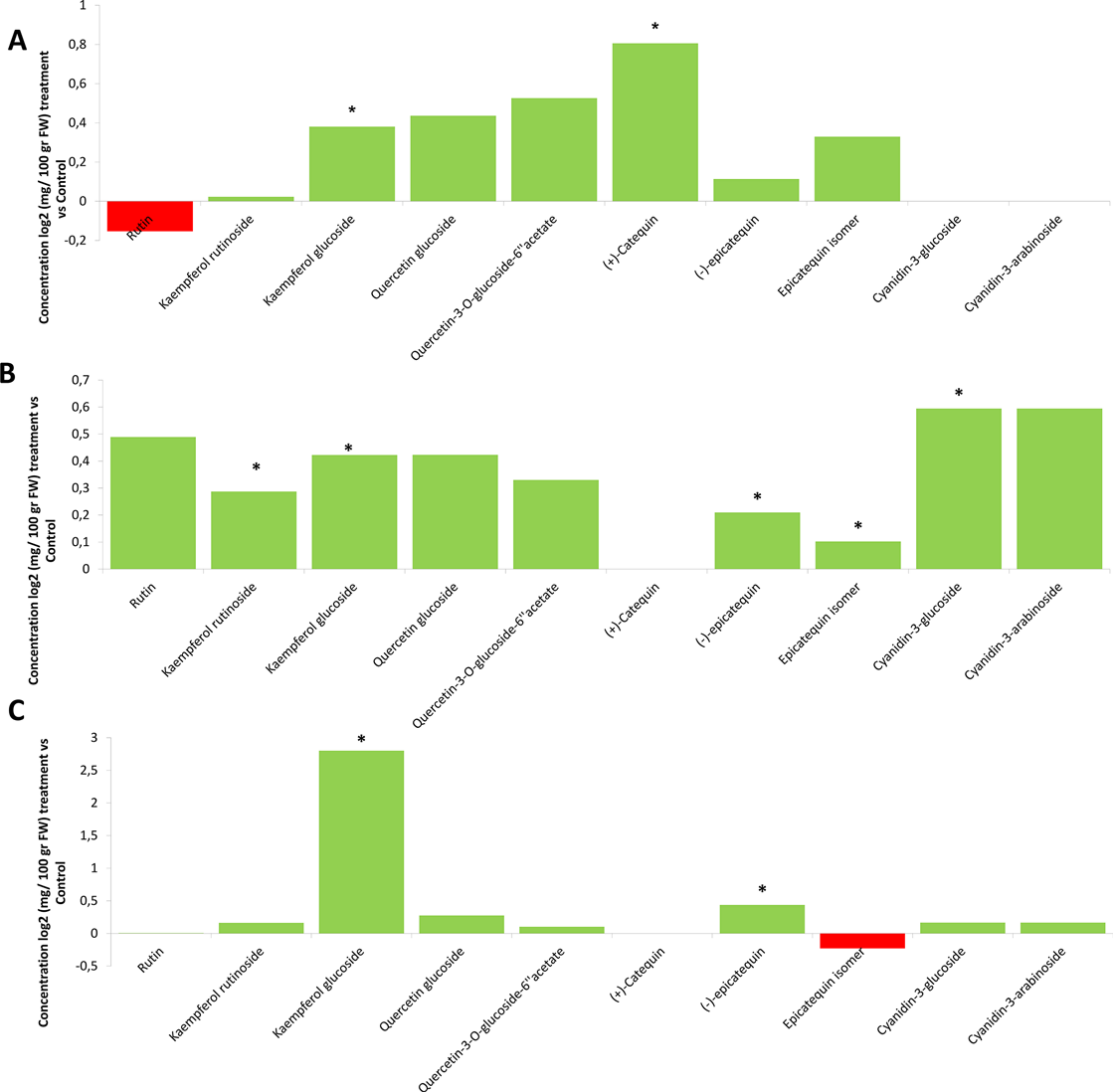
Changes in metabolite concentration (mg per100g FW) on elicited plants over controls in the 3 ripening stages. a)Green stage b) Red stage c) Black stage. An asteriskindicates a significant difference, according to LSD test (p<0.05).

**Fig 7:**
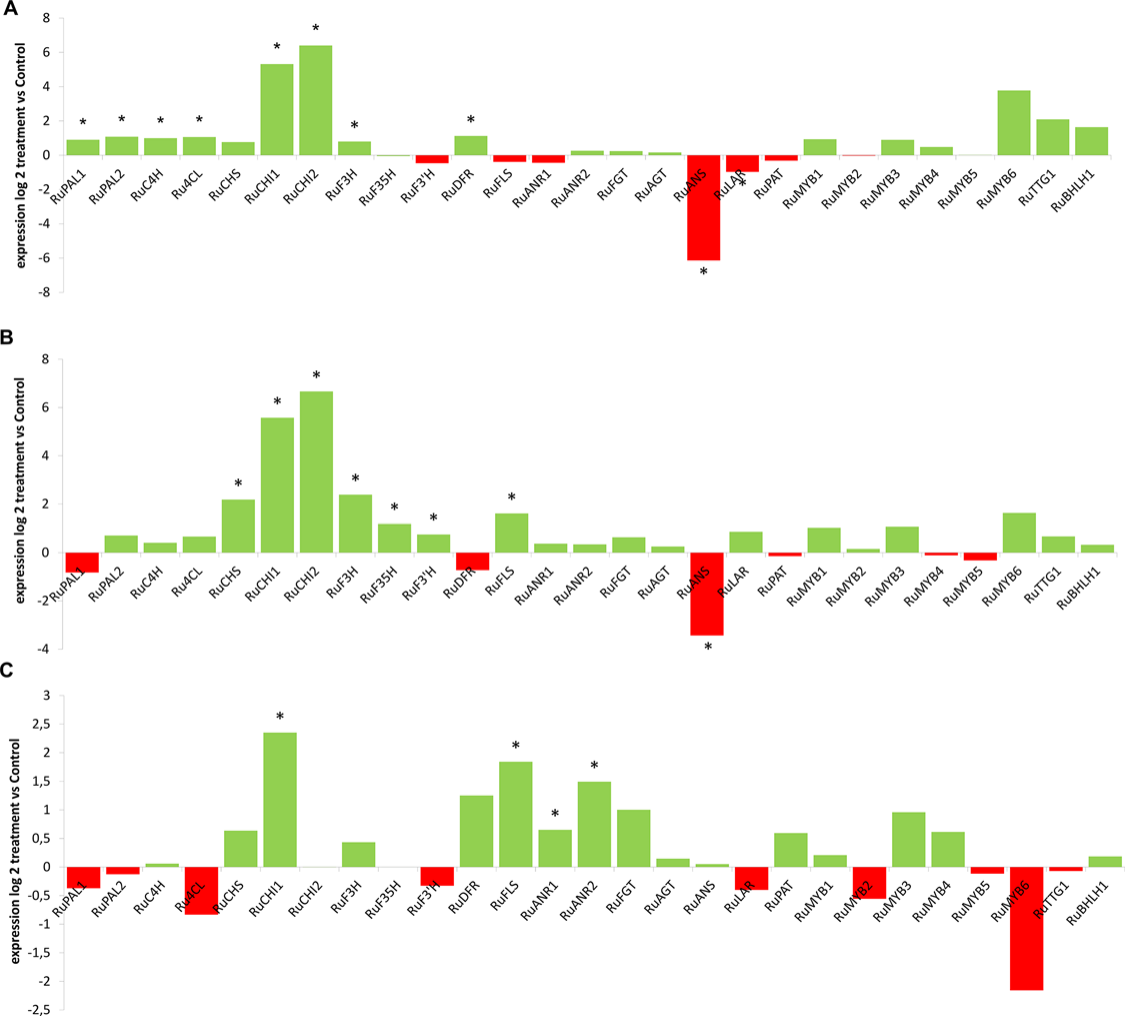
Differential expression of genes involved in the different branches of flavonoid metabolism following PGPR treatment during fruit ripening. A) Changes in gene expression (inoculated compared to untreated) in green fruit. B) Changes in gene expression (inoculated compared to untreated) in red fruit C) Changes in gene expression (inoculated compared to untreated) in ripe, black fruit. An asterix indicates a significant difference, according to LSD test (p<0.05).

We examined whether root inoculation of blackberry with *P*. *fluorescens* N21.4 influenced the expression profiles of metabolites and genes involved in flavonoid biosynthesis in blackberry fruits during ripening (Figs 6 and 7). In green fruit, where the levels of (+)-catechin and kaempferol-glucoside were significantly higher in inoculated plants (Fig 6A), the early genes of the flavonoid biosynthetic pathway (*RuPAL*, *Ru4CH*, *Ru4CL*, *RuCHI* and *RuF3H*) were significantly up-regulated compared to untreated controls (Fig 7A). In red fruit, expression of *RuMYB1*, *RuCHS*, *RuCHI*, *RuF3H*, *RuF3´H*,*RuF3´5´H* and *RuFLS* was higher in PGPR treated fruit (Fig 7B) commensurate with the higher levels of kaempferol-rutinoside, kaempferol-glucoside and epicatechins in treated fruit (Fig 6B). In black fruit, kaempferol-glucoside and epicatechin levels were higher in treated fruit (Fig 6C), consistent with the up-regulation of the genes encoding the enzymes that specifically synthesize these compounds, by elicitation (*RuANR* and *RuFLS*) (Fig 7C). No increase in expression of genes encoding enzymes specific to anthocyanin biosynthesis was observed in PGPR-treated plants, consistent with the absence of any impact of this treatment on anthocyanin levels in ripe fruit.

### PGPR treatment modulates expression of PR genes during fruit ripening

**Fig 8:**
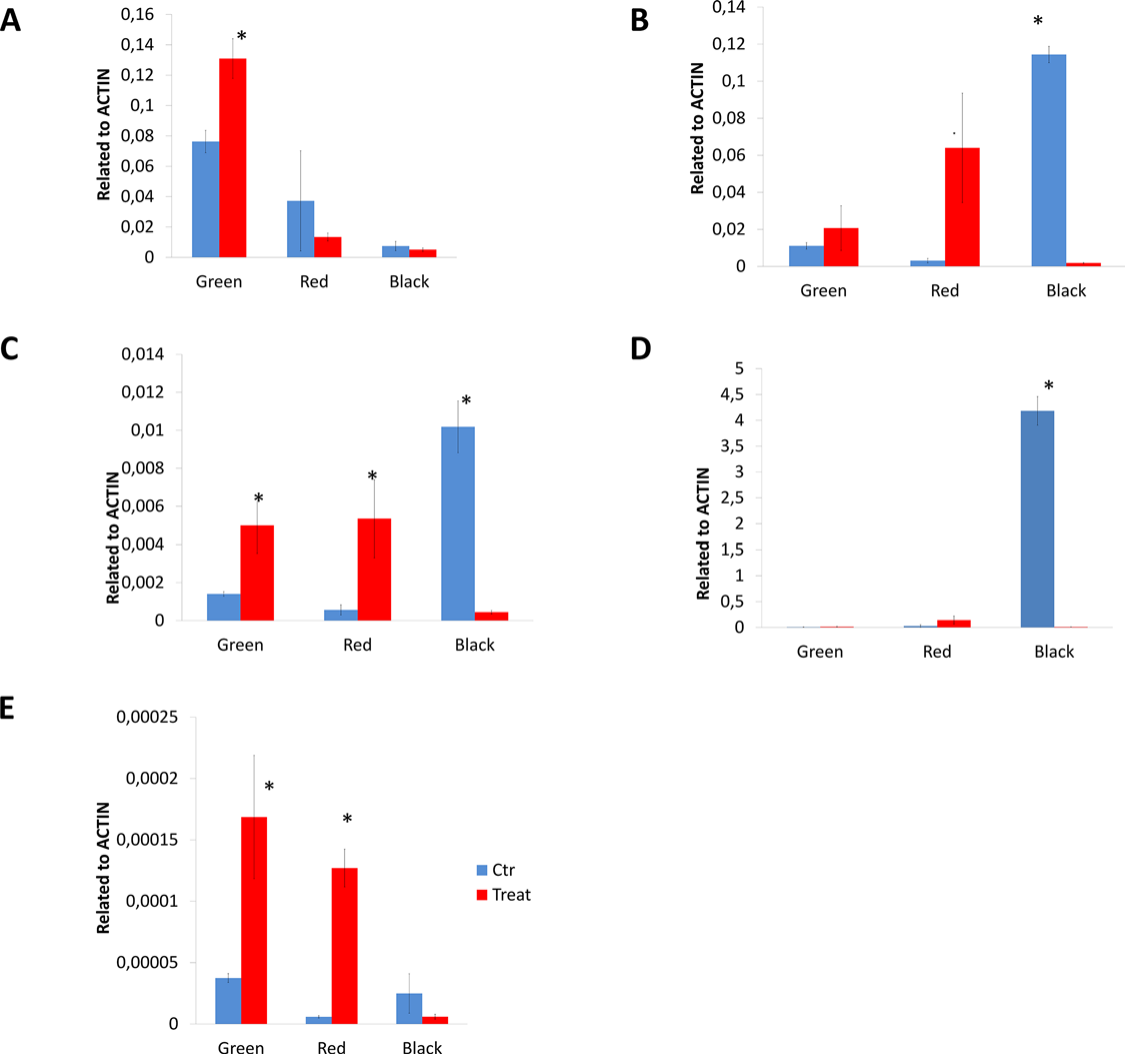
Expression profiles of genes associated with plant microbe interactions in *Rubus* sp. var. Loch Ness fruits at the three development stages (Green, Red and Black) in control (blue bars) and in plants inoculated with *P*. *fluorescens* N21.4 (red bars). a)*RuPR1*, b)*RuPR2*, c)*RuPR3*, d)*RuPR4* e) *RuSBT1*. An asterisk indicates a significant difference between control and inoculated plants according to LSD test (p<0.05).

To characterise further plant responses to PGPR challenge, primers were designed to PR genes identified from sequences of these genes found in the transcriptome of ripe blackberry fruit and annotated by homology to PR genes from other plant species, most notably strawberry [30]. The expression of *ETR1*, *NPR1*, *WRKY3*, *MPK1*, *MPK3* and *MPK4*, was not significantly affected during ripening nor following bacterial challenge (Data not shown). However, genes encoding Pathogen-Related (PR) proteins *RuPR1*, *RuPR2* (β-1,3-glucanase), *RuPR3* (chitinase) and *RuPR4* (unknown function) [40] showed significant differences in expression in inoculate ed plants compared to control plants (Fig 8). In control plants, *RuPR1* transcript levels declined during fruit ripening but *RuPR2*, *RuPR3* and *RuPR4* expression increased throughout fruit maturation, peaking in ripe, black fruit. In plants treated with the rhizobacterium, *RuPR1* showed two-fold higher expression in green fruit of inoculated plants, but not in more mature fruit. *RuPR2* and *RuPR3* were up-regulated in green and red fruit, but down-regulated in black fruit to the same expression level as red untreated fruit; *RuPR4* transcripts increased very significantly in ripe untreated fruit, but this increase was not observed in inoculated ripe fruit. The *ST3*.*3* gene encoding a subtilase protease in *A*. *thaliana* has recently been proposed as an indicator of priming which is the condition of a plant, triggered by PGPR, when it responds faster and/or more strongly in the activation of defence responses when re-challenged by microbial pathogens, herbivorous insects or abiotic stresses[41,42].The profile of the subtilase gene *RuSBT1*, a homologue of *ST3*.*3*, showed elevated transcript levels in green and red fruit from inoculated plants, but levels declined below those of untreated plants in black fruit.

## Discussion

Our results demonstrate that treatment of blackberry plants with *P*. *fluorescens* N21.4 modulates gene expression in fruit of *Rubus* sp. var Loch Ness, affects the profiles of secondary metabolites during fruit ripening, increases flavonol content, and potentially increases nutritional properties of fruit.

Agronomic production of blackberry is expensive, therefore, improving fruit yield, directly associated to kg of fruit per m^2^ produced by the plant, is one of the most important targets for breeders. That has been achieved by treatment with beneficial bacteria. These effects are probably the result of alterations in the balance of plant growth regulators by the bacteria. Although N21.4 is not able to release auxin-like compounds nor degrade 1-aminocyclopropane-1- carboxylic acid in vitro, effects on flowering suggest an interaction with gibberellins involved in the increase in production of flowering stems [43]. Irrespective of the mechanisms responsible for the increase in fruit number, plants need photosynthates for fruiting, therefore, this supply should be supported by an enhanced photosynthetic activity. Improvement of photosynthesis by beneficial bacteria has been reported previously [44], but a decrease in the potential photosynthetic efficiency (Fv/Fm) of blackberry plants treated with N21.4 was associated with higher non-photochemical quenching was observed (Fig 2). This is indicative that PGPR-treated plants may be stressed, there are an systemic response from roots to leaves and therefore it is a good test to check thatan interaction between the plant and the PGPR exists.

In addition to increased fruit yield (Fig 1), higher fruit quality is another target for producers, since these secondary metabolites play significant roles in human health [2]. Although for most genes involved in phenylpropanoid metabolism just one isoform was highly expressed during fruit ripening, some showed more than one highly-expressed isoform suggesting multigene families in blackberry, as in many other species [45,46].

In blackberries, as well as other berries, the contents of flavonols and catechins decrease while anthocyanin contents increase during fruit maturation (Fig 4A) [46]. Overall, the expression of genes in the general phenylpropanoid and flavonol pathways increased during ripening. Expression of genes encoding enzymes of general phenylpropanoid metabolism, such as *RuPAL1*, was higher in the green stage fruit. PAL is considered to be a key regulatory enzyme for flavonoid/anthocyanin biosynthesis during fruit ripening [47,48]. Interestingly, *PAL1* was highly expressed in green stage fruit, whereas *PAL2* was expressed in the black stage, although not as abundantly as *PAL1* (Fig 4B) consistent with data from raspberry and strawberry [49]. Chalcone synthase (CHS) is the enzyme responsible for catalysing the first committed step of the flavonoid biosinthesys pathway. The flavonoid skeleton, synthesized by *CHS*, is converted to chalcones, flavanones, flavonols, anthocyanins, and catechins and also is responsible for other polyketide derivatives, such as benzalacetone and dihydrochalcone, relevant for aroma and colour in other berries [49]. Expression of *CHS* increased during fruit maturation especially in red and black stages, where its expression was almost 10 fold higher than in the green stage (Fig 4B). Also, expression of the genes encoding enzymes in the flavonol pathway, such as *RuF3H* and *RuCHI*, was higher in the black stage fruit. Similarly to *PAL2*, *CHI2* peaked in black stage fruit, showing high expression while *CHI1* predominated in the earlier stages of fruit maturation. Dihydroflavonols are the substrates for several enzymes, that provide precursors of both anthocyanins and flavonols. Therefore, the potential exists for competition between each of these enzymes for common substrates [45]. Lack of specificity for dihydroflavonols and flavanones by flavonoid modification enzymes permits a grid-type pathway, or multiple paths to form the same products [50].

The content of catechins was high at the beginning of berry development and this was consistent with gene expression analysis (Fig 4). The amounts of catechins have been shown to decrease in the progression of ripening in grape (*Vitisvinifera*) and in bilberry[46,51]. It also has been suggested that the presence of catechins in unripe fruits could provide protection against insects/animals (as the taste of catechins is astringent), and increase the resistance to fungal pathogens [52,53]. A strict spatial and temporal control of gene expression ensures the correct accumulation pattern of various secondary products[18].The expression of the gene encoding *RuDFR*, which reduces dihydroflavonols to leucoanthocyanidins (flavan-3, 4-diols), was detected throughout berry development, supplying the biosynthesis of both anthocyanins and catechins from leucoanthocyanidins[54] at the red fruit stage. The expression of anthocyanin pathway genes was specifically up-regulated at the period when anthocyanin accumulation increased most rapidly. Increased expression of *DFR* and *ANS* in red stage fruit was consistent with the onset of anthocyanin formation, and the consistent expression of AGT in red and black stages supports the accumulation of glycosylated anthocyanins at these stages (Fig 4).

Other reports on developmental studies of bilberry, pea (*Pisumsativum*), snapdragon (*Antirrhinum majus*), and petunia (*Petunia hybrida*) flowers [46,55–57] have also shown an apparent inconsistency between low flavonol accumulation in tissues accumulating high levels of anthocyanins and yet high expression of the flavonol biosynthetic genes involved.

All these changes in expression of genes involved in flavonol biosynthesis are controlled by transcription factors (Fig 4C), that control expression of the structural genes of the core pathways. These genes have been identified in many plants and influence the intensity and pattern of anthocyanin biosynthesis. Expression of *RuMYB1*, an homologue of *Rubusidaeus* and *Gerbera* hybrid cv. 'Terra Regina' MYB10 [58,59], increases in the red and black stages consistent with the stimulation in the supply of precursors for anthocyanin accumulation; high expression of *RuMYB6* (a homologue of *MYB7* in *Rosa rugosa* and *GmMYB12B2* in *G*.*max* [38]) in black stage fruit, was consistent with the higher flavonol levels compared to red fruit and the high expression of *RuMYB4* (homologue of *AtMYB4*) [60] in the green stage negatively controlling expression of *C4H*, therefore, controlling flavonol accumulation. Our results suggest the high expression of MYB transcription factors (*RuMYB1-6*, *RuBHLH* and *RuTTG1*) during fruit maturation, plays an important role in fine tuning the phenylpropanoid pathway as previously reported [61]. However, no homologue for *AtMYB12*, was found despite being one of the most important transcription factors controlling flavonol biosynthesis in other plant species [62]. Perhaps a homologue of *AtMYB12* is expressed only in the green fruit stage and the increase in flavonols from red to black fruit is dependent on an alternative MYB regulator, *RuMYB6*.

The systemic effects of N21.4 application, detected on photosynthesis and fruit yield (Fig 1) extends to secondary metabolism in fruits (Figs 5 and 6), confirming our working hypothesis and supporting the idea that elicitation with beneficial bacterial strains improves fruit quality and production [7].N21.4 application affects not only the content of total phenolics and total flavonoids during fruit ripening, but also the profile of different flavonols, catechins and anthocyanins. The metabolic profiles in the three ripening stages (Fig 5) showed that, in general, flavonol levels were higher in elicited fruits during maturation, while catechins and anthocyanidins behaved differently, depending on the stage. In the green stage (Fig 5A), flavonols and catechins were increased by elicitation, and kaempferol-3-glucoside and (+)-catechin were significantly higher, consistent with a significantly higher expression of early genes, from *PAL* to *F3H*, with a remarkable 6-fold higher expression of *CHI* 1 and 2 (Fig 6A); interestingly, *RuMYB6* with a putative role in activating anthocyanin synthesis was significantly expressed more highly, while *ANS* was significantly down-regulated. In the red stage (Fig 5B), kaempferol-rutinoside and kaempferol-glucoside were significantly higher coupled to a significant increase in the expression of *RuMYB1*, with an associated upregulation of genes from *CHS* to *FLS* (Fig 6B); accumulation of anthocyanins and epicatechins. Finally, in the black stage, *CHI2* remained upregulated along with late genes (from *FLS* to *ANR2*) (Fig 6C), associated with significantly higher levels of kaempferol-glucoside and epicatechin (Fig 5C).

In summary, kaempferol-rutinoside and epicatechin were enhanced in edible fruit from N21.4-treated plants, and these effects are, therefore, relevant for health. For example, dietary kaempferol reduces the risk of chronic diseases, especially cancer [63], while (-)-increased epicatechin intake is causally linked to vascular benefits, cancer and protection of the cortisol response [64,65]. This is especially relevant for blackberry, since the consistent increases in bioactive compounds achieved by inoculation with N21.4 in different years predicts a consistent beneficial effect for human health by N21.4 treatment. In addition, recent research has indicated that enrichment of flavonoids can enhance the shelf life performance of fleshy fruit [66,67].The manipulation of flavonoid and catechin biosynthesis in blackberry may be also of importance to enhance shelf life performance of fruit [68].

We also found that expression of several pathogen related proteins was affected by inoculation, although expression of other genes reported to be indicators of ISR or SAR were not affected.

The role of beneficial PGPRs in improving plant fitness by increasing tolerance to pathogens, insect pests, abiotic stress, including drought and salinity has been demonstrated, but very few experiments have been performed in real field conditions to improve fruit quality and the healthiness of these fruits for human consumption [7,69–71]. A consistent increase in fruit yield and quality was observed following inoculation of *P*.*fluorescens* N21.4 in *Rubus* sp. var Loch Ness under field conditions [19,20].

As an approach to understanding the plant response mechanisms following rhizobacterial challenge, the expression of genes involved in plant-microbe interactions have been studied. Upon recognition of MAMPs by pattern recognition receptors (PRRs), a systemic response initiates plant defense mechanisms, involving a number of specific genes, that depend on the specific MAMP and plant considered [72]. The expression of ETR1, NPR1, WRKY3, MPK1, MPK3 and MPK4, or NPR1 was not significantly affected during fruit ripening or by bacterial treatment (data not shown), suggesting that the systemic induction initiated by N21.4 in the plant roots does not trigger the ISR transduction pathway described in *Arabidopsis* at the rosette stage. However, when the Pathogen Related proteins RuPR1, RuPR2 (β-1,3-glucanase), RuPR3 (chitinase) and RuPR4 (unknown direct function but it binds to insoluble chitin and enhances hydrolysis of chitin by other chitinase-like enzymes [40] were analysed, significant differences in expression were found, as well as different behavior depending on the PR gene[47,73,74]. In blackberry control plants, *RuPR1* expression declined during fruit maturation in both control and N21.4-treated plants, unlike in other plants [74], but RuPR2, RuPR3 and RuPR4 increased in their expression throughout fruit maturation to peak at maturity, more than 10 times higher than in green fruit, as also reported for banana and tomato [47,75,76]. Interestingly, in ripe fruit of plants treated with N21.4, expression of RuPR2, RuPR3 and RuPR4 was almost completely lost.

Interestingly, upregulation of *RuPR1*, *RuPR2* and *RuPR3* in green and red fruit suggested that the plants were recognizing the bacterium as a potential threat, at least initially [77–79], and this was consistent with the observed effects on photosynthesis. The down-regulation of *RuPR1*, *RuPR2* and *RuPR3* expression, recorded in black fruit was consistent with reports from other authors,[80] who have suggested that downregulation of PRs could be associated with lowered stress in fruits, which would result in delays to the onset of senescence. However, further studies need to be carried out to support this hypothesis.

Furthermore, another advantage of lowering PR gene expression could be that among the plant allergens listed in the Official Allergen Database of the International Union of Immunological Societies, approximately 25% belong to the group of pathogenesis-related proteins (PR-proteins) [81].

Expression of *RuSBT1*, an homolog of a recently discovered extracellular subtilase from *Arabidopsis*, *SBT3*.*3* [82] probably enhances innate immune responses while loss of its function, likely compromises them. Since *SBT3*.*3* represents a new regulator of primed immunity where epigenetic control has an important role, *RuSBT1* was analysed. During green and red stages its expression was up-regulated, as for all other PRs analysed, indicating that epigenetic control may play a role in the *P*. *fluorescens-Rubus* sp. interaction.

In conclusion, our results demonstrate the coordinated expression of flavonoid biosynthetic genes in relation to the accumulation of anthocyanins, catechins, and flavonols in developing fruits of blackberry. The connection between flavonol, catechins and anthocyanin synthesis in blackberry was evidenced in this study. The differential expression of flavonoid pathway genes in fruits of elicited blackberry plants was shown, demonstrating the physiological mechanisms involved in the improvement in yield and fruit quality under field conditions, and highlighting the genetic targets of elicitation, which can be used to boost production of these compounds in other systems.

## Supporting Information

S1 Fig Isoforms of the genes identified of phenylpropanoid pathway in *Rubus* sp. var. LochNesstranscriptome. Parameters recorded: Gene name, Gene code in transcriptome in the EBI database (accession number: PRJEB6680) (wrote in bold letters the ones that has been analyzed deeply). Gene length, Raw fragment expression and FPKM (Fragments per kb per Million fragments) for (RF1 (Ripe fruit sample 1) and RF2 (Ripe fruit sample 2) (Garcia-Seco et al, 2014),and the score, e-value, and ID for NCBI database. Abbreviations are as follows: phenylalanine ammonia lyase (*PAL*); cinnamate-4-hydroxylase (*C4H*);4-coumaroyl-CoA-ligase (*4CL*); chalcone synthase (*CHS*); chalcone-isomerase (*CHI*); flavanone 3-hydroxylase (*F3H*);flavonoid 3´-hydroxylase (*F3*´*H*); flavonoid 3´5´-hydroxylase (*F3´5´H*); dihydroflavonol 4-reductase (*DFR*); anthocyanidin synthase (*ANS*); flavonol synthase (*FLS*); UDP-glucose: flavonoid 3-O-glucosyltransferase (*FGT*); UDP-glucose: anthocyanidin 3-O-glucosyltransferase (*AGT*); anthocyanidin reductase (*ANR*); leucoanthocyanidin reductase (*LAR*); Putative anthocyanin transporters (*PAT*).
(PDF)

S2 Fig Phylogenetic trees of *RuMYB*. Phylogenetic relationship of RuMYB transcription factors with others MYB that plays a role in the regulation of flavonoid and phenylpropanoid metabolism in *Arabidopsis* and *Fragaria*. An unrooted phylogenetic tree was calculated using the CLUSTALW software using the implemented neighbor-joining method without the function for evolutionary distance correction. Evolutionary distances are proportional to the branch length. Twenty six protein sequences were selected as indicated in the figure.
(PDF)

S1 Table Isoforms of the genes identified of phenylpropanoid pathway in *Rubus* sp. var. Lochnesstranscriptome. Thecolums show per gene, the identification of the gene, gene length, RFKM, Nr-ID, Nr score, Nr-Evalue and the name of the homolog gene.
(PDF)
