# Supplementary figures and images for: Application of Pseudomonas fluorescens to Blackberry under Field Conditions Improves Fruit Quality by Modifying Flavonoid Metabolism
Source: PLoS One. 2015 Nov 11;10(11):e0142639. doi: 10.1371/journal.pone.0142639 (PMC4641737; doi:10.1371/journal.pone.0142639)

1 Supplemental material 2.

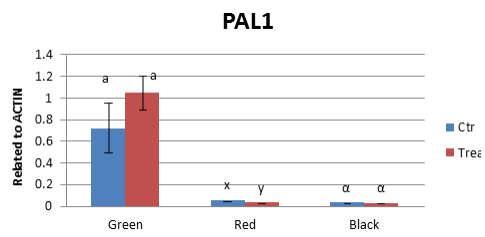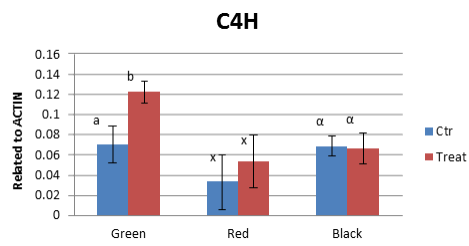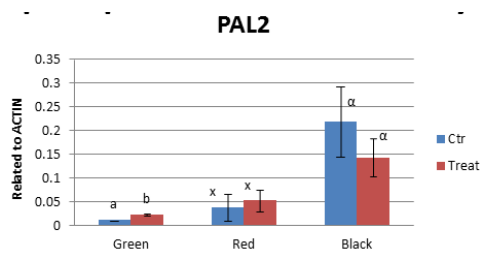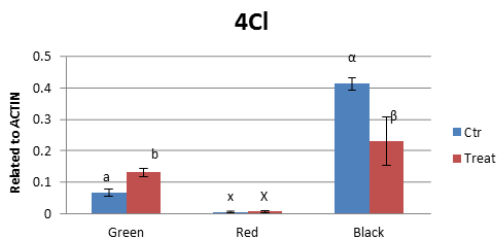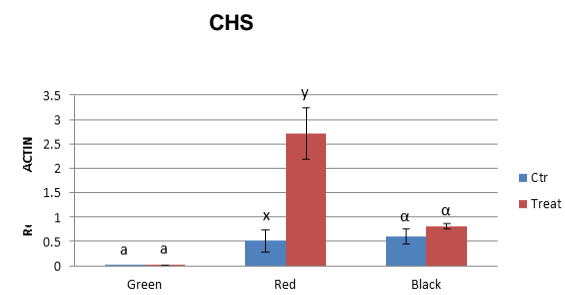

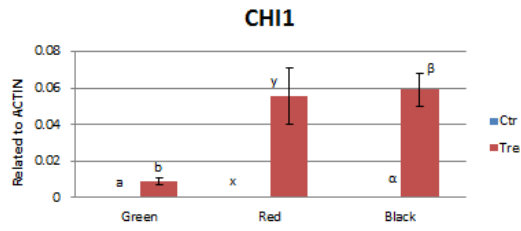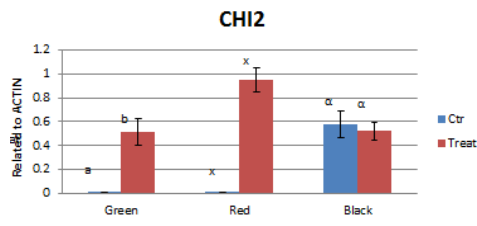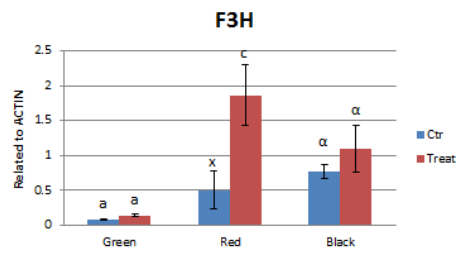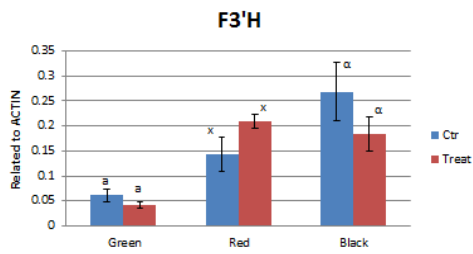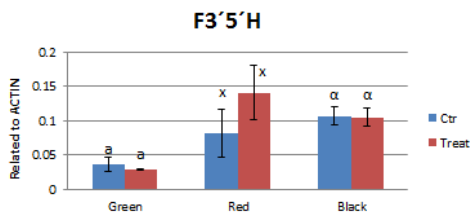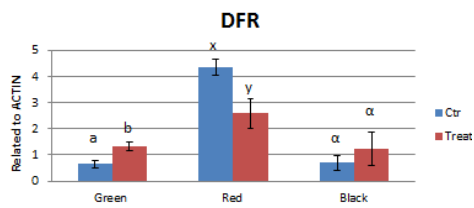

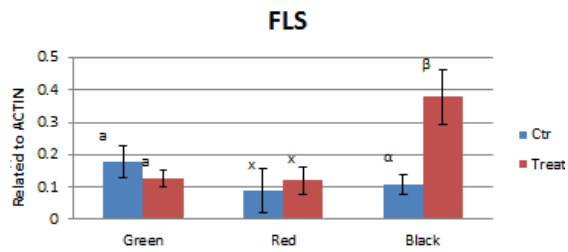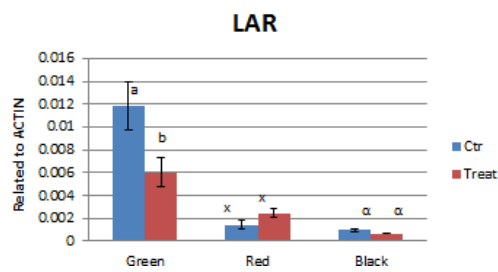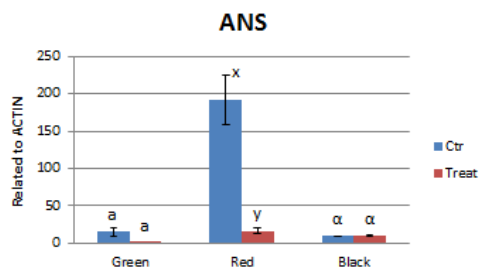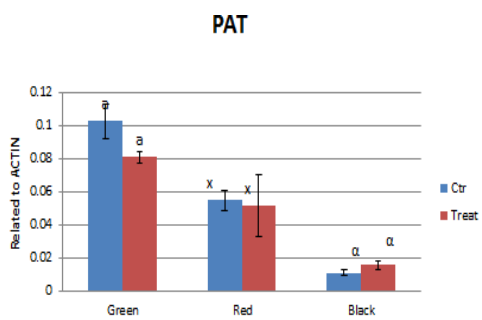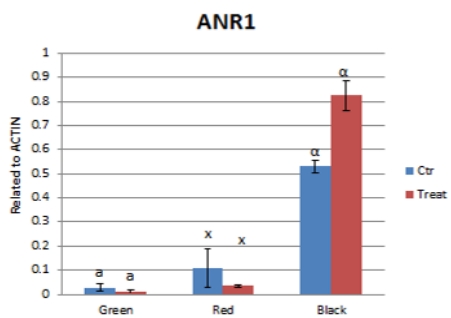

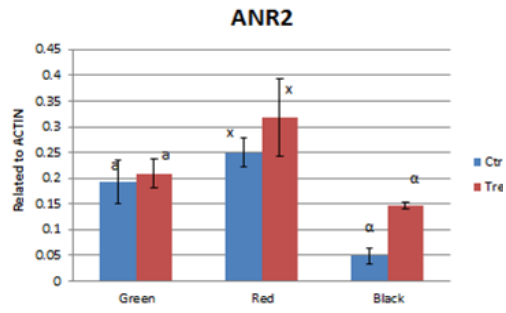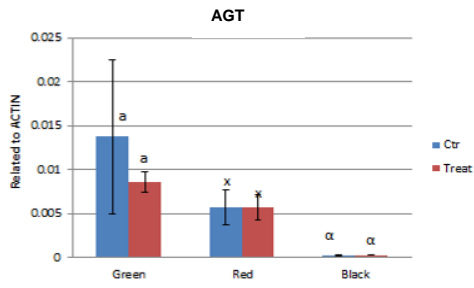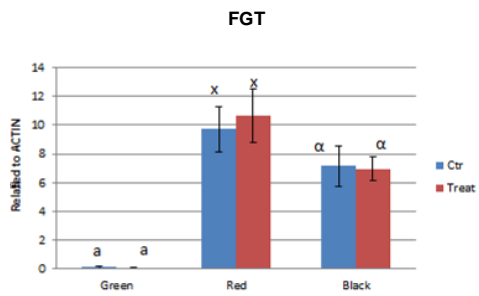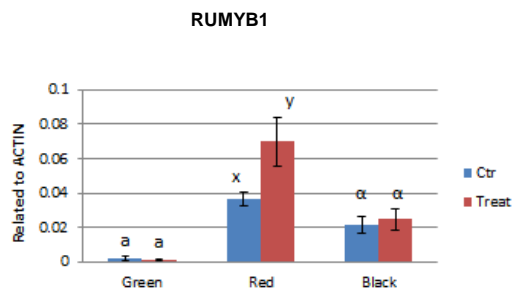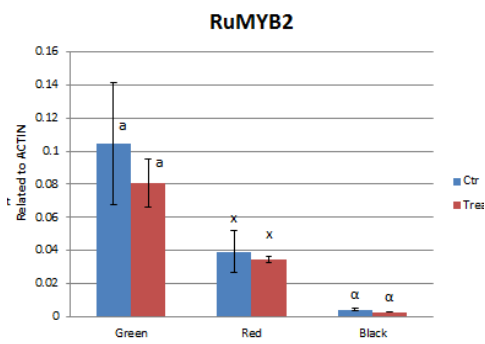

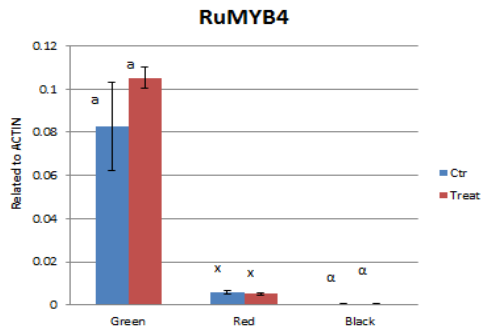

39

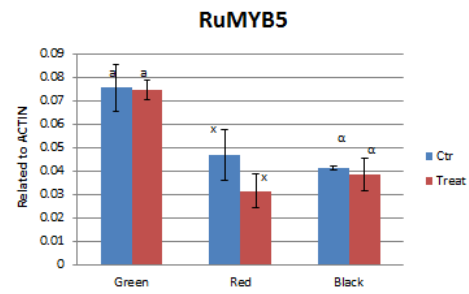

40

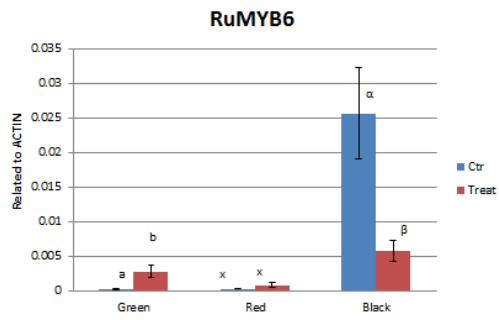

41

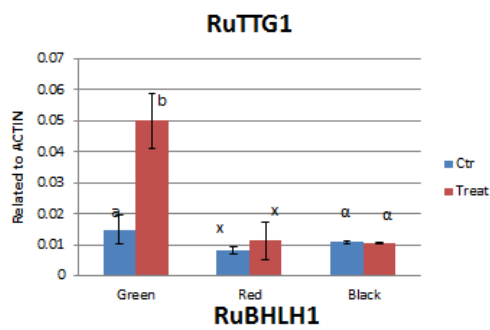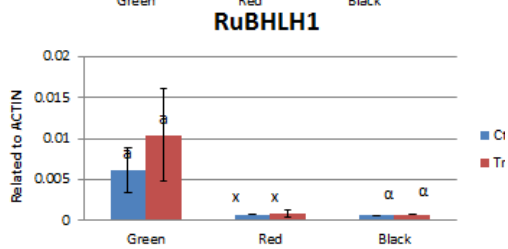

42

43

Supplement: S1 Fig — Parameters recorded: Gene name, Gene code in transcriptome in the EBI database (accession number: PRJEB6680) (wrote in bold letters the ones that has been analyzed deeply). Gene length, Raw fragment expression and FPKM (Fragments per kb per Million fragments) for (RF1 (Ripe fruit sample 1) and RF2 (Ripe fruit sample 2) (Garcia-Seco et al, 2014),and the score, e-value, and ID for NCBI database. Abbreviations are as follows: phenylalanine ammonia lyase (PAL); cinnamate-4-hydroxylase (C4H);4-coumaroyl-CoA-ligase (4CL); chalcone synthase (CHS); chalcone-isomerase (CHI); flavanone 3-hydroxylase (F3H);flavonoid 3´-hydroxylase (F3´H); flavonoid 3´5´-hydroxylase (F3´5´H); dihydroflavonol 4-reductase (DFR); anthocyanidin synthase (ANS); flavonol synthase (FLS); UDP-glucose: flavonoid 3-O-glucosyltransferase (FGT); UDP-glucose: anthocyanidin 3-O-glucosyltransferase (AGT); anthocyanidin reductase (ANR); leucoanthocyanidin reductase (LAR); Putative anthocyanin transporters (PAT). (PDF) [file pone.0142639.s001.pdf]
