# Supplementary material for: Application of Pseudomonas fluorescens to Blackberry under Field Conditions Improves Fruit Quality by Modifying Flavonoid Metabolism
Source: PLoS One. 2015 Nov 11;10(11):e0142639. doi: 10.1371/journal.pone.0142639 (PMC4641737; doi:10.1371/journal.pone.0142639)
Supplement: S2 Fig — Phylogenetic relationship of RuMYB transcription factors with others MYB that plays a role in the regulation of flavonoid and phenylpropanoid metabolism in Arabidopsis and Fragaria. An unrooted phylogenetic tree was calculated using the CLUSTALW software using the implemented neighbor-joining method without the function for evolutionary distance correction. Evolutionary distances are proportional to the branch length. Twenty six protein sequences were selected as indicated in the figure. (PDF) [file pone.0142639.s002.pdf]

Supplemental material 3.

Phylogenetic relationship of RuMYB transcription factors with others MYB that plays a role in the regulation of flavonoid and phenylpropanoid metabolism in *Arabidopsis* and *Fragaria*. An unrooted phylogenetic tree was calculated using the CLUSTALW software using the implemented neighbor-joining method without the function for evolutionary distance correction. Evolutionary distances are proportional to the branch length. Twenty six protein sequences were selected as indicated in the figure**.**

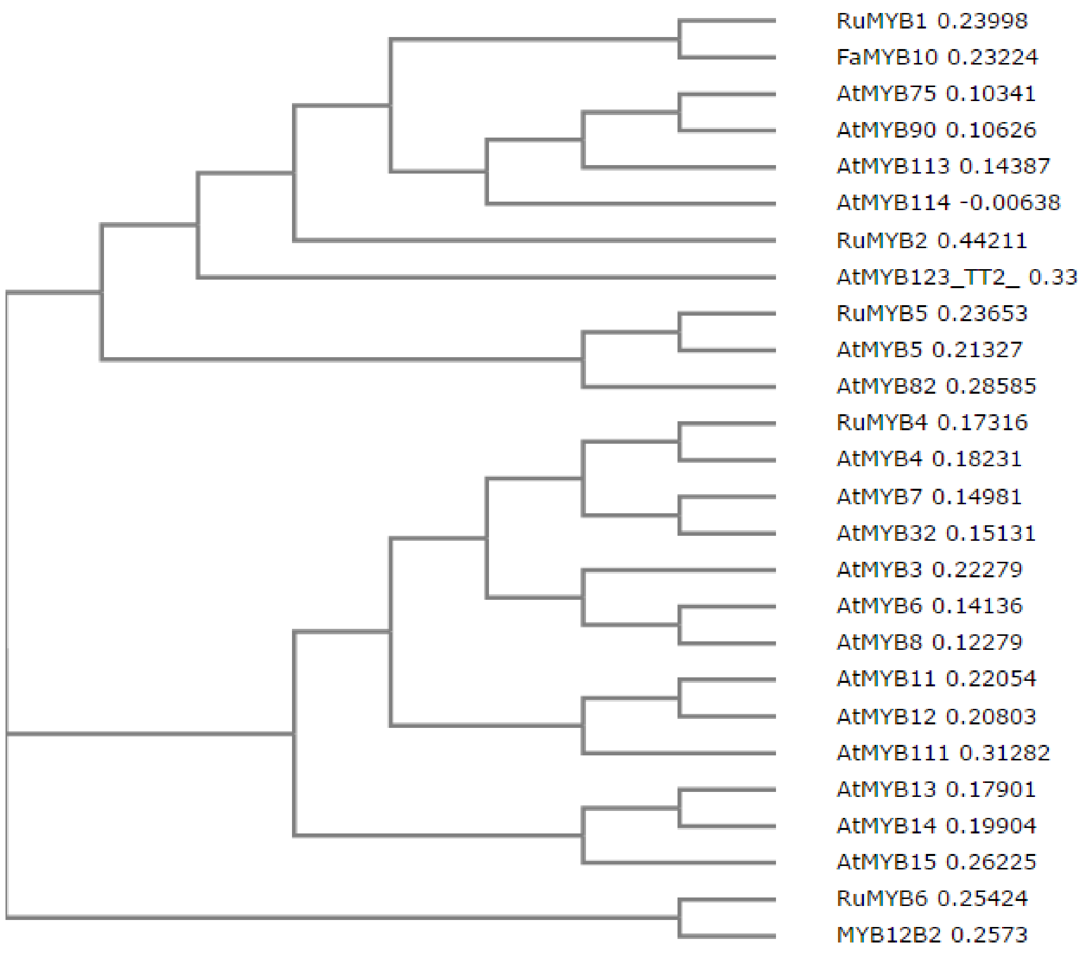

>RuMYB1

MEVRKGAWTKEEDHLLRNYIEKHGEGRWHKVPLEAGLNRCRKSCRMRWLNYLKPNIKR
GDFAEDEVDLMIRLRKLLGNRWSLIAGRLPGRTSNDVKNYWSARRRRKIDFGVPKDNEP
PKITKTTIIRPRPRTFTKSLHHLSAEAATSMHSSEQNNSWPSSSPPIENGIDEWKTLMLED
VFTNFWVEDVASLTGTGVNSAEQGFETDLWHFLQEEAR

>FaMYB10

MEGFGVRKGAWTKEEDELLKQFIEIHGEGKWHHVPLKSGLNRCRKSCRLRWLNYLKPNI
KRGEFAEDEVDLIIRLHKLLGNRWSLIAGRLPGRTANDVKNYWNTYQRKKDQKTASYAKK
LKVKPRENTIAYTIVRPRPRTFIKRFNFTERYANIEHNHSEVSYTSSLPTEPPQTLQLENVT
DWWKDFSEDSTESIDRTMCSGLGLEDHDFFTNFWVEDMLLSASNDLVNISYV

>RuMYB2

MGRGPRCSKDGLNKGAWMATEDKLLLDYIKNHGEGKWSNVAKETGSHFLLNLINYFVNL
FLLVFFWKKGLIHYEHGVGLVHKLACWFFIFTTSRSIIVRWNMYVGLKRCGKSCRLRWMN
YLRPDIRRGSISDDEEELIVRLHKLLGNRYSHRSLINLPFFVNCNLSHVNLINLQMVSDSRE
TSWANRQNQELLELQFSENGTSSKFIPVMQCHCQNHSQYRHSTAAHSYSSWYCANCGS
SPKRRLLCGHKFRRLGQSCFSFTNVQTRLPMPINNYYWGWGSFRKFAHEFHDGFQHSG
FLQDTGLLCKLEQCHGPWRSWCSQRSHFSTTADDHGQECSSCFWVLGFISGVGLAWES
KCHAELISFLSVLSPIFIYIHVLQCLLLVLKKKKKVLVDAWP

>AtMYB123(TT2)

MGKRATTSVRREELNRGAWTDHEDKILRDYITTHGEGKWSTLPNQAGLKRCGKSCRLR
WKNYLRPGIKRGNISSDEEELIIRLHNLLGNRWSLIAGRLPGRTDNEIKNHWNSNLRKRLP
KTQTKQPKRIKHSTNNENNVCVIRTKAIRCSKTLLFSDLSLQKKSSTSPLPLKEQEMDQGG
SSLMGDLEFDFDRIHSEFHFPDLMDFDGLDCGNVTSLVSSNEILGELVPAQGNLDLNRPF
TSCHHRGDDEDWLRDFTC

>RuMYB4

MGRSPCCEKAHTNKGAWTKEEDDRLIAYIRAHGEGCWRSLPKAAGLLRCGKSCRLRWI
NYLRPDLKRGNFTEEEDELIIKLHSLLGNKWSLIAGRLPGRTDNEIKNYWNTHIRRKLLTRG
IDPATHRPLNETAQDSATTTTISFAASSAIKEEDQKISTSIGIVGKESINPVQEKCPDLNLEL
RISPPSQTKPVESFKSGGRGVCFSCSLGLKDSKSCCSCGMDNIGPTSAGTSNIAYDFLGL
KNGVLDYRSLEMK

>AtMYB4

MGRSPCCEKAHTNKGAWTKEEDERLVAYIKAHGEGCWRSLPKAAGLLRCGKSCRLRWI
NYLRPDLKRGNFTEEEDELIIKLHSLLGNKWSLIAGRLPGRTDNEIKNYWNTHIRRKLINRG
IDPTSHRPIQESSASQDSKPTQLEPVTSNTINISFTSAPKVETFHESISFPGKSEKISMLTFK
EEKDECPVQEKFPDLNLELRISLPDDVDRLQGHGKSTTPRCFKCSLGMINGMECRCGRM
RCDVVGGSSKGSDMSNGFDFLGLAKKETTSLLGFRSLEMK

>RuMYB5

MRNPSSSASASKGKSSAAAAAASASAPAVVSSSSSKTPCCVKVGLKRGPWTPEEDELLA
NYIKKEGEGRWRTLPKRAGLLRCGKSCRLRWMNYLRPSVKRGQIAPDEEDLILRLHRLLG
NRURPSUQHTYTYIHIYDSIPFGDSGUGLRLQCFYWIDDHDDDESVFIYRWSLIAGRIPGR
TDNEIKNYWNTHLSKKLISQGIDPRTHKPLNPDSSSGDHAAVNGASSSKTNQHRGMSSN
PNPKSPITSSSPVSTEEAAAPNARENGYLDQGNANDDLGVIVHGAGGYGNGMTSNDSD

GSAPAMGALRSNLLLGNEDDEDINCCTDDVFSSFLNSLINEDPFAAAAAAAGQHQLQPLQ
LVQQPNGIAPSSDLSAPAFGGWESAVIMSSAFIQKDPHRVNVDQTQ

>AtMYB5

MMSCGGKKPVSKKTTPCCTKMGMKRGPWTVEEDEILVSFIKKEGEGRWRSLPKRAGLL
RCGKSCRLRWMNYLRPSVKRGGITSDEEDLILRLHRLLGNRWSLIAGRIPGRTDNEIKNY
WNTHLRKKLLRQGIDPQTHKPLDANNIHKPEEEVSGGQKYPLEPISSSHTDDTTVNGGDG
DSKNSINVFGGEHGYEDFGFCYDDKFSSFLNSLINDVGDPFGNIIPISQPLQMDDCKDGIV
GASSSSLGHD

>RuMYB6

MDVKPGRVSNTIRSSHEEEEDDVMMDLRRGPWTVEEDLALMNYIANHGEGRWNSLARC
AGLKRTGKSCRLRWLNYLRPDVRRGNITLEEQLLILELHSRWGNRWSKIAQHLPGRTDN
EIKNYWRTRVQKHAKQLKCDVNSKQFKDTMRYLWMPRLVERIQAAASSATASTAASSSIT
TTITTAPNPSYHFNNNFQSTGAGHVALQQATLIGSNNDLAASYTTPENSSTGASSDSFGN
QVSPVSELTDYYTTMSVNNNNPSPMEYFQAPNPHHQIGYRDSMTSPSGYVFNQGGLNS
HSFQAASAPEQNNNGQWGMDGGDFSDNLWNVEESDMWFLQQQLGNI

>MYB12B2

MERSLSGSSEEEMSITKGPWTEEEDSVLFNYITVHGEGHWNSVARYTGLKRTGKSCRLR
WLNYLRPNVRRGNITLEEQLLILDLHSRWGNRWSKIAEHLPGRTDNEIKNYWRTRVVKQA
EQLKCEVNSKQFRDTLRFVWMPRMLERIQAQASSSSSSSSGQAHQTTLISNAQAHSDPC
GVMTTNPSLLSEVSSSALDAHAPSLYDSSASYNLMGGGSSSSYSAEYCYYSDIAGNGFG
GSDLWTDENICFLQQLLADHDLS

>AtMYB3

MGRSPCCEKAHMNKGAWTKEEDQLLVDYIRKHGEGCWRSLPRAAGLQRCGKSCRLRW
MNYLRPDLKRGNFTEEEDELIIKLHSLLGNKWSLIAGRLPGRTDNEIKNYWNTHIKRKLLS
RGIDPNSHRLINESVVSPSSLQNDVVETIHLDFSGPVKPEPVREEIGMVNNCESSGTTSEK
DYGNEEDWVLNLELSVGPSYRYESTRKVSVVDSAESTRRWGSELFGAHESDAVCLCCRI
GLFRNESCRNCRVSDVRTH

>AtMYB6

MGRSPCCEKAHTNKGAWTKEEDQRLVDYIRNHGEGCWRSLPKSAGLLRCGKSCRLRWI
NYLRPDLKRGNFTDDEDQIIIKLHSLLGNKWSLIAGRLPGRTDNEIKNYWNTHIKRKLLSHG
IDPQTHRQINESKTVSSQVVVPIQNDAVEYSFSNLAVKPKTENSSDNGASTSGTTTDEDL
RQNGECYYSDNSGHIKLNLDLTLGFGSWSGRIVGVGSSADSKPWCDPVMEARLSLL

>AtMYB7

MGRSPCCEKEHMNKGAWTKEEDERLVSYIKSHGEGCWRSLPRAAGLLRCGKSCRLRWI
NYLRPDLKRGNFTHDEDELIIKLHSLLGNKWSLIAARLPGRTDNEIKNYWNTHIKRKLLSKG
IDPATHRGINEAKISDLKKTKDQIVKDVSFVTKFEETDKSGDQKQNKYIRNGLVCKEERVV

VEEKIGPDLNLELRISPPWQNQREISTCTASRFYMENDMECSSETVKCQTENSSSISYSSIDISSSNVGYDFLGLKTRILDFRSLEMK

>AtMYB8

MGRSPCCEKAHMNKGAWTKEEDQRLIDYIRNHGEGSWRSLPKSVGLLRCGKSCRLRWINYLRPDLKRGNFTDGEEQIIVKLHSLFGNKWSLIAGKLPGRTDNEIKNYWNTHIKRKLLNRGIDPKTHGSIIEPKTTSFHPRNEDLKSTFPGSVKLKMETSCENCASTSGTTTDEDLRLSVDCDYRYDHLDKELNLDLTLGYSPTRFVGVGSCY

>AtMYB11

MGRAPCCEKVGIKKGRWTAEEDRTLSDYIQSNGEGSWRSLPKNAGLKRCGKSCRLRWINYLRSDIKRGNITPEEEDVIVKLHSTLGTRWSTIASNLPGRTDNEIKNYWNSHLSRKLHGYFRKPTVANTVENAPPPPKRRPGRTSRSAMKPKFILNPKNHKTPNSFKANKSDIVLPTTTIENGEGDKEDALMVLSSSSLSGAEEPGLGPCGYGDDGDCNPSINGDDGALCLNDDIFDSCFLLDDSHAVHVSSCESNNVKNSEPYGGMSVGHKNIETMADDFVDWDFVWREGQTLWDEKEDLDSVLSRLLDGEEMESEIRQRDSNDFGEPLDIDEENKMAAWLLS

>AtMYB12

MGRAPCCEKVGIKRGRWTAEEDQILSNYIQSNGEGSWRSLPKNAGLKRCGKSCRLRWINYLRSDLKRGNITPEEEELVVKLHSTLGNRWSLIAGHLPGRTDNEIKNYWNSHLSRKLHNFIRKPSISQDVSAVIMTNASSAPPPPQAKRRLGRTSRSAMKPKIHRTKTRKTKKTSAPPEPNADVAGADKEALMVESSGAEAELGRPCDYYGDDCNKNLMSINGDNGVLTFDDDIIDLLLDESDPGHLYTNTTCGGDGELHNIRDSEGARGFSDTWNQGNLDCLLQSCPSVESFLNYDHQVNDASTDEFIDWDCVWQEGSDNNLWHEKENPDSMVSWLLDGDDEATIGNSNCENFGEPLDHDDESALVAWLLS

>AtMYB13

MGRRPCCEKIGLKKGPWSAEEDRILINYISLHGHPNWRALPKLAGLLRCGKSCRLRWINYLRPDIKRGNFTPHEEDTIISLHQLLGNRWSAIAAKLPGRTDNEIKNVWHTHLKKRLHHSQDQNNKEDFVSTTAAEMPTSPQQQSSSSADISAITTLGNNNDISNSNKDSATSSEDVLAIIDESFWSEVVLMDCDISGNEKNEKKIENWEGSLDRNDKGYNHDMEFWFDHLTSSSCIIGEMSDISEF

>AtMYB14

MGRAPCCEKMGVKRGPWTPEEDQILINYIHLYGHSNWRALPKHAGLLRCGKSCRLRWINYLRPDIKRGNFTPQEEQTIINLHESLGNRWSAIAAKLPGRTDNEIKNVWHTHLKKRLSKNLNNGGDTKDVNGINETTNEDKGSVIVDTASLQQFSNSITTFDISNDNKDDIMSYEDISALIDDSFWSDVISVDNSNKNEKKIEDWEGLIDRNSKKCSYSNSKLYNDDMEFWFDVFTSNRRIEEFSDIPEF

>AtMYB15

MGRAPCCEKMGLKRGPWTPEEDQILVSFILNHGHSNWRALPKQAGLLRCGKSCRLRWM
NYLKPDIKRGNFTKEEEDAIISLHQILGNRWSAIAAKLPGRTDNEIKNVWHTHLKKRLEDYQ
PAKPKTSNKKKGTKPKSESVITSSNSTRSESELADSSNPSGESLFSTSPSTSEVSSMTLIS
HDGYSNEINMDNKPGDISTIDQECVSFETFGADIDESFWKETLYSQDEHNYVSNDLEVAG
LVEIQQEFQNLGSANNEMIFDSEMDFWFDVLARTGGEQDLLAGL

>AtMYB32

MGRSPCCEKDHTNKGAWTKEEDDKLISYIKAHGEGCWRSLPRSAGLQRCGKSCRLRWI
NYLRPDLKRGNFTLEEDDLIIKLHSLLGNKWSLIATRLPGRTDNEIKNYWNTHVKRKLLRK
GIDPATHRPINETKTSQDSSDSSKTEDPLVKILSFGPQLEKIANFGDERIQKRVEYSVVEER
CLDLNLELRISPPWQDKLHDERNLRFGRVKYRCSACRFGFGNGKECSCNNVKCQTEDS
SSSSYSSTDISSSIGYDFLGLNNTRVLDFSTLEMK

>AtMYB75

MEGSSKGLRKGAWTTEEDSLLRQCINKYGEGKWHQVPVRAGLNRCRKSCRLRWLNYLK
PSIKRGKLSSDEVDLLLRLHRLLGNRWSLIAGRLPGRTANDVKNYWNTHLSKKHEPCCKI
KMKKRDITPIPTTPALKNNVYKPRPRSFTVNNDCNHLNAPPKVDVNPPCLGLNINNVCDN
SIIYNKDKKKDQLVNNLIDGDNMWLEKFLEESQEVDILVPEATTTEKGDTLAFDVDQLWSL
FDGETVKFD

>AtMYB82

MECKREEGKSYVKRGLWKPEEDMILKSYVETHGEGNWADISRRSGLKRGGKSCRLRWK
NYLRPNIKRGSMSPQEQDLIIRMHKLLGNRWSLIAGRLPGRTDNEVKNYWNTHLNKKPNS
RRQNAPESIVGATPFTDKPVMSTELRRSHGEGGEEESNTWMEETNHFGYDVHVGSPLP
LISHYPDNTLVFDPCFSFTDFFPLL

>AtMYB90

MEGSSKGLRKGAWTAEEDSLLRLCIDKYGEGKWHQVPLRAGLNRCRKSCRLRWLNYLK
PSIKRGRLSNDEVDLLLRLHKLLGNRWSLIAGRLPGRTANDVKNYWNTHLSKKHESSCCK
SKMKKKNIISPPTTPVQKIGVFKPRPRSFSVNNGCSHLNGLPEVDLIPSCLGLKKNNVCEN
SITCNKDDEKDDFVNNLMNGDNMWLENLLGENQEADAIVPEATTAEHGATLAFDVEQLW
SLFDGETVELD

>AtMYB111

MGRAPCCEKIGLKRGRWTAEEDEILTKYIQTNGEGSWRSLPKKAGLLRCGKSCRLRWIN
YLRRDLKRGNITSDEEEIIVKLHSLLGNRWSLIATHLPGRTDNEIKNYWNSHLSRKIYAFTA
VSGDGHNLLVNDVVLKKSCSSSSGAKNNNKTKKKKGRTSRSSMKKHKQMVTASQCFS
QPKELESDFSEGGQNGNFEGESLGPYEWLDGELERLLSSCVWECTSEEAVIGVNDEKV
CESGDNSSCCVNLFEEEQGSETKIGHVGITEVDHDMTVEREREGSFLSSNSNENNDKD
WWVGLCNSSEVGFGVDEELLDWEFQGNVTCQSDDLWDLSDIGEITLE

>AtMYB113

MGESPKGLRKGTWTTEEDILLRQCIDKYGEGKWHRVPLRTGLNRCRKSCRLRWLNYLKP
SIKRGKLCSDEVDLVLRLHKLLGNRWSLIAGRLPGRTANDVKNYWNTHLSKKHDERCCK
TKMINKNITSHPTSSAQKIDVLKPRPRSFSDKNSCNDVNILPKVDVVPLHLGLNNNYVCES
SITCNKDEQKDKLININLLDGDNMWWESLLEADVLGPEATETAKGVTLPLDFEQIWARFD
EETLELN

>AtMYB114

MEGSSKGLRKGAWTAEEDSLLRQCIGKYGEGKWHQVPLRAGLNRCRKSCRLRWLNYLK
PSIKRGKFSSDEVDLLLRLHKLLGNRWSLIAGRLPGRTANDVKNYWNTHLSKKHEPCCKT
KIKRINIITPPNTPAQKVDIF
