# Supplementary material for: Application of Pseudomonas fluorescens to Blackberry under Field Conditions Improves Fruit Quality by Modifying Flavonoid Metabolism
Source: PLoS One. 2015 Nov 11;10(11):e0142639. doi: 10.1371/journal.pone.0142639 (PMC4641737; doi:10.1371/journal.pone.0142639)
Supplement: S1 Table — Thecolums show per gene, the identification of the gene, gene length, RFKM, Nr-ID, Nr score, Nr-Evalue and the name of the homolog gene. (PDF) [file pone.0142639.s003.pdf]

Supplemental material 1. Isoforms of the genes identified of phenylpropanoid pathway in *Rubus* sp. var. Lochness transcriptome.

	geneID	Gene Length	RF1 FPKM	RF1 FPKM	Nr-ID	Nr-Score	Nr-Evalue	
Ru4CL	Unigene12367	794	0,08	0,80	gi|9651917|gb|AAF91310.1|AF239687_1	517,7	8,00E-146	4-coumarate:coA ligase 1 [Rubus idaeus]
	**Unigene16366**	**2093**	**47,71**	**27,18**	**gi|9651915|gb|AAF91309.1|AF239686_1**	**1055,4**	**0**	**4-coumarate:coA ligase 2 [Rubus idaeus]**
	Unigene13214	311	3,62	1,43	gi|9651913|gb|AAF91308.1|AF239685_1	140,2	9,00E-33	4-coumarate:coA ligase 3 [Rubus idaeus]
	Unigene13466	332	0,80	1,15	gi|470134772|ref|XP_004303212.1|	186	1,00E-46	PREDIC.: 4-coumarate--CoA ligase 1-like [Fragaria vesca subsp. vesca]
	Unigene4142	1732	2,94	4,22	gi|9651913|gb|AAF91308.1|AF239685_1	939,5	0	4-coumarate:coA ligase 3 [Rubus idaeus]
RuAGT	**Unigene8869**	**1735**	**1858,04**	**1503,45**	**gi|157888994|dbj|BAF80946.1|**	**774,6**	**0**	**UDP-glucose: anthocyanin 3-glucosylltransferase [Rosa hybrid cultivar]**
RuANR	**Unigene2512**	**1341**	**4,69**	**5,93**	**gi|110564477|gb|ABG76842.1|**	**625,5**	**6E-178**	**anthocyanidin reductase [Fragaria x ananassa]**
	**Unigene2522**	**1339**	**23,64**	**23,21**	**gi|470129936|ref|XP_004300863.1|**	**578,6**	**8,00E-164**	**PREDIC.: anthocyanidin reductase-like [Fragaria vesca subsp. vesca]**
	Unigene653	627	1,69	2,53	gi|297745410|emb|CBI40490.3|	51,2	3,00E-42	unnamed protein product [Vitis vinifera]
RuANS	**Unigene4439**	**1677**	**2021,14**	**1848,25**	**gi|470125461|ref|XP_004298720.1|**	**710,3**	**0**	**PREDIC.: leucoanthocyanidin dioxygenase-like [Fragaria vesca subsp. vesca]**
	Unigene7051	1422	247,88	297,89	gi|462419517|gb|EMJ23780.1|	605,1	9,00E-172	hypothetical protein PRUPE_ppa008455mg [Prunus persica]
	Unigene10888	1576	134,12	84,78	gi|470144031|ref|XP_004307670.1|	685,6	0	PREDIC.: flavonol synthase/flavanone 3-hydroxylase-like [Fragaria vesca
RuC4H	Unigene19458	238	0,00	0,27	gi|470127623|ref|XP_004299767.1|	159,1	2,00E-38	PREDIC.: trans-cinnamate 4-monooxygenase-like [Fragaria vesca subsp. vesca]
	Unigene21546	293	0,00	0,00	gi|462422005|gb|EMJ26268.1|	196,1	1,00E-49	hypothetical protein PRUPE_ppa018282mg, partial [Prunus persica]
	Unigene21762	203	0,33	0,00	gi|470127623|ref|XP_004299767.1|	134,4	5,00E-31	PREDIC.: trans-cinnamate 4-monooxygenase-like [Fragaria vesca subsp. vesca]
	**Unigene5281**	**1878**	**305,35**	**278,87**	gi|221328921|gb|ACM17896.1|	1001,5	0	cinnamate-4-hydroxylase [Rubus occidentalis]
	Unigene9503	376	0,00	0,68	gi|470127623|ref|XP_004299767.1|	233,8	6,00E-61	PREDIC.: trans-cinnamate 4-monooxygenase-like [Fragaria vesca subsp. vesca]
RuCHI	**CL5405 (Iso1)**	**1061**	**467.8972**	**417.0408**	**gi|470144168|ref|XP_004307734.1|**	**397.1**	**2.00E-109**	**PREDIC.: chalcone--flavonone isomerase-like [Fragaria vesca subsp. vesca]**
	**Unigene16160 (Iso2)**	**1556**	**428.1638**	**429.4765**	**gi|470143579|ref|XP_004307451.1|**	**407.1**	**4.00E-112**	**PREDIC.: chalcone--flavonone isomerase 1-like [Fragaria vesca subsp. vesca]**
	Unigene17871	1063	3.6753	4.0663	gi|470116014|ref|XP_004294183.1|	470.3	2.00E-131	PREDIC.: chalcone--flavonone isomerase 2-like [Fragaria vesca subsp. vesca]
RuCHS	**CL6637**	**1712**	**1024,76**	**939,12**	**gi|22086372|gb|AAM90652.1|AF400567_1**	**775**	**0**	**chalcone synthase 6 [Rubus idaeus]**
RuDFR	**Unigene5062**	**1117**	**407,39**	**408,88**	**gi|470111242|ref|XP_004291858.1|**	**645,2**	**0**	**PREDIC.: dihydroflavonol4-reductase/flavanone 4-reductase- [Fragaria vesca]**
	CL2114	1421	7,64	8,59	gi|470142458|ref|XP_004306924.1|	549,3	6,00E-155	PREDIC.: dihydroflavonol-4-reductase-like [Fragaria vesca subsp. vesca]
	Unigene1168	1638	22,48	20,53	gi|470142462|ref|XP_004306926.1|	489,2	9,00E-137	PREDIC.: dihydroflavonol-4-reductase-like [Fragaria vesca subsp. vesca]
	Unigene10789	1389	10,49	13,59	gi|470116315|ref|XP_004294328.1|	619,8	3,00E-176	PREDIC.: dihydroflavonol-4-reductase-like [Fragaria vesca subsp. vesca]
	CL3922	1472	0,22	0,22	gi|470109675|ref|XP_004291118.1|	618,6	8,00E-176	PREDIC.: putative dihydroflavonol-4-reductase-like [Fragaria vesca subsp. vesca]
	Unigene12257	877	1,21	1,88	gi|462416254|gb|EMJ20991.1|	264,6	1,00E-69	hypothetical protein PRUPE_ppa026509mg, partial [Prunus persica]
	Unigene10163	1272	4,48	5,60	gi|470112433|ref|XP_004292439.1|	672,5	0	PREDIC.: epimerase family protein slr1223-like [Fragaria vesca subsp. vesca]
RuF3´5´H	**Unigene3145**	**1596**	**173,76**	**155,73**	**gi|470103987|ref|XP_004288406.1|**	**446**	**8E-124**	**PREDIC.: uncharacterized protein LOC101310934 isoform 1 [Fragaria vesca sub**

	CL1570	1656	2,36	3,99	gi|470125387|ref|XP_004298684.1|	846,7	0	PREDIC.: flavonoid 3'5';-hydroxylase-like [Fragaria vesca subsp. vesca]
RuF3H	**Unigene16038**	**1850**	**2162,83**	**1975,59**	**gi|161597781|gb|ABX74780.1|**	**717,2**	**0**	**flavanone-3-hydroxylase [Rubus sp. SSL-2007]**
	Unigene18040	1285	2,06	6,28	gi|470145288|ref|XP_004308273.1|	620,2	2,00E-176	PREDIC.: naringenin,2-oxoglutarate3-dioxygenase-like[Fragaria vesca subsp. vesca]
	Unigene2612	1495	14,88	11,40	gi|470144544|ref|XP_004307914.1|	650,2	0	PREDIC.: flavanone 3-dioxygenase-like [Fragaria vesca subsp. vesca]
	Unigene1033	1286	22,50	19,57	gi|470141150|ref|XP_004306298.1|	641,7	0	PREDIC.: naringenin,2-oxoglutarate3-dioxygenase-like [Fragaria vesca subsp. vesca]
	Unigene10888	1576	134,12	84,78	gi|470144031|ref|XP_004307670.1|	685,6	0	PREDIC.: flavonol synthase/flavanone3-hydroxylase-like isoform 1 [Fragaria vesca]
	Unigene7051	1422	247,88	297,89	gi|462419517|gb|EMJ23780.1|	605,1	9,00E-172	hypothetical protein PRUPE_ppa008455mg [Prunus persica]
	CL430	1545	3,77	4,16	gi|462403437|gb|EMJ08994.1|	497,3	3,00E-139	hypothetical protein PRUPE_ppa025000mg, partial [Prunus persica]
RuF3´H	CL1193	**1262**	**1,26**	**2,07**	gi|462401212|gb|EMJ06769.1|	610,5	1E-173	hypothetical protein PRUPE_ppa008740mg [Prunus persica]
	**Unigene3117**	**1782**	**312,81**	**327,42**	**gi|377550336|dbj|BAL63027.1|**	**906,7**	**0**	**flavonoid 3';-hydroxylase [Fragaria x ananassa]**
	CL1570	907	**1,17**	**1,05**	gi|470125387|ref|XP_004298684.1|	476,5	2,00E-133	PREDIC.: flavonoid 3';,5';-hydroxylase-like [Fragaria vesca subsp. vesca]
		1656	**2,36**	**3,99**	gi|470125387|ref|XP_004298684.1|	846,7	0	PREDIC.: flavonoid 3';,5';-hydroxylase-like [Fragaria vesca subsp. vesca]
	CL1859	1745	**6,45**	**3,72**	gi|470149050|ref|XP_004310057.1|	831,2	0	PREDIC.: geraniol 8-hydroxylase-like [Fragaria vesca subsp. vesca]
	CL5325	2145	**1,36**	**2,61**	gi|470105227|ref|XP_004288988.1|	905,2	0	PREDIC.: flavonoid 3';-monooxygenase-like [Fragaria vesca subsp. vesca]
RuFLS	**Unigene7051**	**1422**	**247,88**	**297,89**	**gi|462419517|gb|EMJ23780.1|**	**605,1**	**9,00E-172**	**hypothetical protein PRUPE_ppa008455mg [Prunus persica]**
RuFGT	**CL2933**	**1283**	**1,29**	**1,59**	**gi|157888996|dbj|BAF80947.1|**	**664,1**	**0**	**UDP-glucose:flavonol 3-glucosyltransferase [Rosa hybrid cultivar]**
RuLAR	**Unigene488**	**1313**	**4,14**	**6,00**	**gi|401715671|gb|AFP99288.1|**	**646,4**	**0**	**leucoanthocyanidin reductase [Fragaria x ananassa]**
RuPAL	**CL4350 PAL 1**	**1725**	**0,61**	**0,41**	**gi|14195012|sp|Q9M568.1|PAL1_RUBID**	**1002,3**	**0**	**RecName: Full=Phenylalanine ammonia-lyase 1; AltName: Full=RiPAL1**
	**Unigene698 PAL 2**	**803**	**8,99**	**7,84**	**gi|14195012|sp|Q9M568.1|PAL1_RUBID**	**369,4**	**3,00E-101**	**RecName: Full=Phenylalanine ammonia-lyase 1; AltName: Full=RiPAL1**
RuPAT	**Unigene7392**	**1794**	**4.4662**	**4.3936**	**gi|470144854|ref|XP_004308062.1|**	**880.2**	**0**	**PREDIC.: protein TRANSPARENT TESTA 12-like [Fragaria vesca subsp. vesca]**
	Unigene7484	1447	0.4119	0.5272	gi|470144854|ref|XP_004308062.1|	734.2	0	PREDIC.: protein TRANSPARENT TESTA 12-like [Fragaria vesca subsp. vesca]
	Unigene5791	1690	1.4106	1.5045	gi|470144854|ref|XP_004308062.1|	876.7	0	PREDIC.: protein TRANSPARENT TESTA 12-like [Fragaria vesca subsp. vesca]
RuMYB1	CL343	1079	12,70	11,43	gi|393808395|gb|AFN25407.1|	427,2	2E-118	R2R3 MYB transcription factor [Rubus hybrid cultivar]
RuMYB2	Unigene13073	1156	19,42	19,69	gi|315937167|gb|ADU56173.1|	464,9	1E-129	MYB domain class transcription factor [Fragaria vesca]
RuMYB4	**CL4055**	**1182**	**6,83**	**9,52**	**gi|470107055|ref|XP_004289866.1|**	**470,7**	**2,00E-131**	**PREDIC.: myb-related protein 308-like [Fragaria vesca subsp. vesca]**
RuMYB5	**CL216**	**2127**	**2.8642**	**2.8391**	**gi|470117735|ref|XP_004295005.1|**	**509.6**	**8.00E-143**	**PREDIC.: transcription repressor MYB5-like [Fragaria vesca subsp. vesca]**
RuMYB6	**Unigene12151**	**1391**	**8.5213**	**9.0939**	**gi|327412615|emb|CCA29096.1|**	**624**	**1.00E-177**	**putative MYB transcription factor [Rosa rugosa]**
RuBHLH1	**Unigene12758**	**2842**	**26,93**	**28,41**	**gi|401782370|dbj|BAM36702.1|**	**1261,9**	**0**	**bHLH transcriptional factor AN1 homolog [Rosa hybrid cultivar]**

TTG1	**CL6588**	**1557**	**16,12**	**13,84**	**gi|336442437|gb|AEI55401.1|**	**703,7**	**0**	**TTG1 [Rubus idaeus]**
RuPR1	**Unigene9504**	**387**	**0,34**	**0,49**	**gi|470120663|ref|XP_004296417.1|**	**250**	**8,00E-66**	**PREDIC.: pathogenesis-related protein PR-1-like [Fragaria vesca subsp. vesca]**
RuPR2	**Unigene6519**	**1305**	**7,81**	**6,19**	**gi|16903144|gb|AAL30426.1|AF435089_1**	**538,5**	**9,00E-152**	**beta-1,3-glucanase [Prunus persica]**
RuPR3	**Unigene13345**	**330**	**0,60**	**0,39**	**gi|470144783|ref|XP_004308027.1|**	**208,8**	**2,00E-53**	**PREDIC.: acidic endochitinase-like [Fragaria vesca subsp. vesca]**
RuPR4	**Unigene8680**	**465**	**1,85**	**2,05**	**gi|470114870|ref|XP_004293631.1|**	**271,2**	**3,00E-72**	**PREDIC.: pathogenesis-related protein PR-4B-like [Fragaria vesca subsp. vesca**
